# Injuries and Associated Factors in Swedish Sporting and Utility Trial Dogs—A Cross-Sectional Study

**DOI:** 10.3390/ani14030398

**Published:** 2024-01-26

**Authors:** Ann Essner, Catarina Kjellerstedt, Amie L. Hesbach, Helena Igelström

**Affiliations:** 1 IVC Evidensia Djurkliniken Gefle, Norra Gatan 1, SE-803 21 Gävle, Sweden; 2 Department of Women’s and Children’s Health, Uppsala University Hospital, Uppsala University, SE-751 85 Uppsala, Sweden; 3 Veterinär Catarina Kjellerstedt AB, Östgötavägen 5, SE-186 35 Vallentuna, Sweden; 4 EmpowerPhysio, Maynard, MA 01754, USA

**Keywords:** sports, utility dogs, working dogs, injury, agility, obedience, rally obedience, tracking, protection, search

## Abstract

**Simple Summary:**

Canine sporting competitions, e.g., agility, obedience, rally obedience, and utility trials, e.g., protection, tracking, search, and messenger, are physically and mentally demanding disciplines. There is a growing concern within the dog sports community that some disciplines and breeds may be at higher injury risk. This study aimed to describe the frequencies and types of injuries experienced amongst Swedish sporting and utility trial dogs and to explore associations between discipline, breed, sex, neuter status, age, and injury history. Through a questionnaire survey, information about 1582 dogs was collected. During their lifetime, more than half of the dogs (*n* = 928, 58.7%) had suffered from an injury whether it appeared during training, competition, or outside of sport. Key findings were that muscular, joint, and dermatologic injuries were most commonly reported and the most common injury locations were the thoracic, lumbar and lumbosacral spine, paw, head, shoulder, and stifle. In a multivariate analysis, Border Collies, Belgian Malinois, and higher age at the time of report increased the odds of injury history. Our results provide more understanding of injuries in sporting and utility dogs and contribute to injury prevention strategies that potentially enhance canine welfare.

**Abstract:**

Canine sporting competitions, e.g., agility, obedience, rally obedience, and utility trials, e.g., protection, tracking, search, and messenger, are physically and mentally demanding disciplines. This study aimed to describe the types and frequencies of injuries experienced amongst Swedish sporting and utility trial dogs and to explore associations between discipline, breed, sex, neuter status, age, and injury history. Dog handlers provided information on competition-level dogs (*n* = 1582) through a cross-sectional survey. The overall proportion of dogs sustaining any injury during their lifetime, whether it was during competition, training, or outside of sport, was 58.7% (*n* = 928). Muscular, joint, and dermatologic injuries were most commonly reported and the most common injury locations were the thoracic, lumbar and lumbosacral spine, paw, head, shoulder, and stifle. According to multivariate analysis, Border Collie (adjusted OR 1.93), Belgian Malinois (adjusted OR 2.51) and higher age at the time of report (adjusted OR 1.81–9.67) increased the odds of injury history. Our results provide more understanding of injuries in sporting and utility dogs and contribute to injury prevention strategies that potentially enhance canine welfare.

## 1. Introduction

Obedience, rally obedience, agility, and utility dog trials are canine sporting and trial disciplines consisting of progressive competitive levels, testing the physical and mental capacities, workability, and performance of the dogs. In dog sports and utility trials, handlers navigate the dogs through various tasks, e.g., heelwork, obstacles of various heights to overcome, objects of various weights to retrieve, searching and tracking for people in the forest, running and jumping to catch, and adapting and withstanding environmental challenges [1]. The obstacles are hurdle jumps of pre-determined heights and the objects are of various pre-determined sizes and weights in relation to the dog’s height at the withers [2,3,4,5]. In the utility disciplines, dogs perform scent tasks, e.g., tracking and searching for hidden people, and long-distance running between stations from one handler to another (messenger) [3]. The dogs’ success depends on their ability to jump, retrieve objects, perform scent tasks, focus on precision movement during heelwork, i.e., the dog’s shoulder remaining level with the handler’s leg throughout various maneuvers, and to complete tasks without faults according to a judge and dog sport-specific regulations [6,7]. The physical and mental requirements vary among disciplines, where disciplines such as agility, obedience, and utility involve tasks that require muscle strength and power, while rally obedience has a lower physical impact [1,7,8]. Utility disciplines, like tracking, searching and messenger, demand cardiorespiratory and muscular endurance from the dog [9,10,11]. All disciplines consist of progressive levels, from beginner to higher competitive levels. Each level consists of increasingly challenging exercises, e.g., heavier dumbbells, recalls with quicker stops, higher obstacles, longer tracks, and larger search areas in heavier terrain [2,3,4,5].

Sporting and utility trial competitions are popular within the canine industry and veterinary and rehabilitation professionals are regularly consulted to evaluate, treat, and provide rehabilitation interventions to these dogs. Therefore, increased awareness and expanded information regarding injury characteristics, including onset of injury and associated factors in sporting and utility trial dogs participating in various disciplines, are essential for veterinary and rehabilitation professionals to be effective within this field, and for dog handlers, judges, breeders, and organizers of training, trials, and competitions to possibly prevent increased incidence of injury [1,12].

While training towards competition in obedience, rally obedience, agility, and utility trials provides opportunities for physical activity and mental enrichment, it is not without associated risks. Behavior-based selection of dogs for participation in utility trials and breeding programs has been applied for many years [13,14]. One trait, boldness, has especially been found useful in predicting performance in utility trial dogs, i.e., high-performing dogs are bolder compared to low-performing dogs [15,16]. Positive correlations have been reported between boldness and playfulness, exploratory behavior and a pro-social attitude, and negative correlations with avoidance and inhibition behavior [17,18,19]. Personality traits in the dog may be a risk factor for injury independent of breed, but this has, to the knowledge of the authors, not been evaluated in the literature. The identification and understanding of risk factors for sports-related injuries in dogs are essential for the well-being of canine athletes and their long-term participation in these activities. Over the last decade, the identification of associated factors concerning canine health and well-being has gained more attention among dog handlers and animal health care professionals [20,21,22,23,24,25,26,27,28]. There is also a concern about these increased risks of injury in sporting and utility trial dogs and concerns surrounding possible welfare implications, which may result in ethical dilemmas for stakeholders, i.e., dog handlers, judges, breeders, and organizers of competitions [29,30].

Based on previous canine epidemiological research, the prevalence rate of injuries in competitive agility dogs varies with the population surveyed. Prevalence rates between 8.0 and 45.5% have been found in dogs suffering at least one injury during their career [24,31,32,33,34,35]. A recent survey of working dogs presented an injury proportion of 45.5%, which is higher than in competitive agility dogs [24,31,32]. Interestingly, musculoskeletal injuries in working dogs showing temporary but overt signs of injury at onset never reoccurred in 92.0% of the cases [31]. Unlike in the field of human sports medicine and rehabilitation, the return to participation, sports, and the performance continuum [36], and the prognosis for a continued competitive career in sporting and utility trial dogs are still largely unknown due to a paucity of data [37]. In two previous studies of agility dogs returning to sports following orthopedic injury and cranial cruciate ligament rupture, two-thirds of the dogs returned to competition [38,39]. Nearly half of the dogs participated at a lower level of performance [38].

Dogs practicing agility have been more extensively studied. There are several factors associated with injury described in the literature. Among breeds, Border Collies have repeatedly been reported to have a higher risk of agility-related injuries [27,32,40]. In working utility dogs, Belgian Malinois and Labrador Retrievers have been associated with higher injury risk compared to German Shepherd Dogs [41]. Another study in working utility dogs did not find any breed association with traumatic dentoalveolar injury [42]. Studies indicate higher proportions of injury history with increasing age of the dog at the time of survey participation [28,34,40,41,43]. Altered sexual status has been addressed as a potential risk factor for some canine cancers and joint disorders [44]. There is, however, no consensus regarding sexual statuses and relationships to injury in competitive dogs. Spay and neuter have been associated with hip dysplasia, elbow dysplasia, and cranial cruciate ligament disease in certain breeds, albeit not in the context of sports or utility [44]. Regarding sports, increased injury proportions have been reported in spayed female agility dogs [34,45], while the results in another study do not indicate any relationships between altered sexual status and injuries in agility dogs [40]. Besides sport-specific characteristics, the probability of injury has been shown to vary with geographic location [40]. This may be a reflection of several cultural differences which may influence injury incidence. For example, it has been reported that the duration of daily walks is longer in Finnish and Swedish agility dogs compared to in the USA [46,47,48], and the sexual statuses are unaltered to a larger extent in Swedish competition dogs compared to American competition dogs [32,47,49].

Studies on the risk of and protective factors to injury continue to be scarce, especially in utility dogs, and consequently, preventive advice to dog handlers, e.g., physical activities, activity-to-rest ratio, and sport-specific training, are limited and continue to be based on experience.

This study aimed to describe frequencies and any types of injuries experienced in Swedish sporting and utility trial dogs participating in various disciplines and from different breeds, and to explore associations between discipline, breed, sex, neuter status, age, and any injury history. Based on previous studies in agility dogs and the experience of the authors, we hypothesized that increased injury proportions are associated with dogs participating in agility, Border Collies, altered sexual status, and higher age at the time of injury report. We hypothesized that dogs’ sex is not associated with injury history.

## 2. Materials and Methods

### 2.1. Study Design

This study was descriptive and correlational with a cross-sectional study design, exploring the injury frequencies and relationships between disciplines, breeds and type, sex, neuter status, the dogs’ age at report, and comprehensive injury history in a sample of Swedish sporting and utility trial dogs.

### 2.2. Dogs and Data Collection

This study was conducted with competitive sporting and utility trial dogs (*n* = 1582) using reported data from an online questionnaire survey completed by dog handlers [29,47]. The questionnaire examined the demographic variables of the dog, e.g., sex, age, body weight, breed, and sports-related variables, e.g., participation in various disciplines, health history, and return to competition. Items regarding a specific injury complaint leading to the dog handler’s and/or medical attention during the lifespan of the dog were included in the survey. Respondents reported complaints of injury, confirmed by a veterinarian and/or self-diagnosed. They were asked about the presence of injuries, if the injury(-ies) kept the dog from participating in sport-specific training, competition and/or other physical activity, and about the onset of the injury(-ies). The respondents were then asked to describe the injury(-ies) and whether the injury(-ies) had been confirmed by a veterinarian or not. Details of the questionnaire survey have been previously published [47]. Variables regarding demographics and health history are described in the Supplementary file, Table S1.

### 2.3. Definition of Injuries

In the present study, musculoskeletal, neurological, dermatological, dental, and ocular injuries were defined as injuries regardless of whether they represented acute injury or acute-on-chronic repetitive trauma. Any injury, despite the onset of injury signs appearing as a result of activity (including non-sporting activity) or directly as a result of engaging in sports-related activity, was included. The presence of canine hip and elbow dysplasia has been reported previously and was not defined as injury in this study [47]. Reported injury complaints and musculoskeletal, neurological, dermatological, dental, and ocular conditions were reviewed and classified according to Pyramidion (Svensk Djursjukvård, Stockholm, Sweden) [50], a diagnostic coding system with a hierarchical structure, used in the veterinary patient record and practice management system Provet Cloud© (Nordhealth Ltd., Helsinki, Finland, 2023). According to the diagnostic coding system, data from reported injury were categorized into an organ system (e.g., the digestive system), a level of that organ system (e.g., oral cavity, throat, esophagus), and the type of injury or disease process (e.g., traumatic), and, when possible, to a specific diagnosis (e.g., tooth fracture). To enable comparison with other previously reported injury research in sporting and utility dogs, injuries were also categorized and described by canine anatomical body locations [32]. Injury distributions were reported once per dog by body system in the Pyramidion diagnosis system and once by canine anatomical body location. Unspecified signs of injury were categorized into the multiorgan category in the Pyramidion diagnosis system, e.g., lameness and signs of pain. Unspecified injury locations were categorized as “forelimb”, “hindlimb”, or “limb” when possible. In the case in which no anatomical body location was further specified by the respondent, the injury location was assigned to the type of tissue involved, i.e., bone, joint, muscle, pain, soft tissue, or tendon.

A dog was categorized into the injury group if the respondent had answered “Yes” to the question if the dog had had any injury and/or if the respondent reported injury complaints and veterinary diagnosis in succeeding survey questions. An audit of all cases revealed that a few (*n* = 37) respondents had answered “No” to the question if the dog had had an injury and they then reported injury complaints and veterinary diagnosis. These dogs had musculoskeletal conditions, e.g., osteoarthritis and spondylosis, and were included in the injury group since chronic degenerative changes may present with acute signs of injury.

### 2.4. Statistical Methods

Categorical variables were summarized as frequencies and proportions (%). The distributions of continuous variables were manually inspected. Mean and standard deviation (SD) was calculated for normally distributed variables and median and interquartile range (IQR) for non-normally distributed variables.

The distributions of different types of injuries are expressed as frequencies and proportions in the total cohort, by participation in various sporting and utility trial disciplines and by the most common breeds and type of dogs. Individual breeds, except mixed breeds, are presented in the results if there were ≥50 dogs in the sample of that breed. Dogs from other pure breeds were collapsed into a group called “Other purebreds”. The injury proportions are expressed as the frequencies and proportions of the number of dogs in individual disciplines, breeds and type, sex, neuter status (intact/altered), and the dogs’ age at the time of report. To avoid small frequencies in the contingency tables, individual disciplines were included in the analysis of injury history if there were ≥50 dogs in the sample participating in that discipline. Utility trial disciplines including mondioring were collapsed into one group “Utility” and further combined into four categories, i.e., protection, tracking, search, and messenger [3,47].

Chi-squared tests were used to test for differences in injury frequency distribution in disciplines, breeds, sex, neuter status, age category at the time of report, and any injury history. The 95% Wilson confidence intervals of the injury proportions were estimated using Epipools [51]. Sensitivity analysis was performed to assess the possible influence of veterinary-confirmed diagnosis in reported frequencies of injury history by participation in disciplines and breeds. In the sensitivity analysis, we excluded all self-diagnosed injuries that, according to the respondent, were not confirmed by a veterinarian.

A binary multivariate logistic regression model was used to account for differences in lifetime exposure for different aged dogs at the time of report and to estimate the associations between the binary outcome variable, i.e., any injury history, and independent variables. Associations to injury history were identified by entering all the independent variables, i.e., competing in obedience, rally obedience, agility and/or utility trial, breed or type, sex, neuter status, and age at report, into the regression model simultaneously, i.e., forced entry. To check for multicollinearity between the independent variables, the variance inflation factor (VIF) was calculated. The VIF for the independent variables was acceptable (range 1.004–1.567), suggesting there was no multicollinearity.

All statistical analyses were performed with SPSS Statistics 26.0 (IBM Corp., Armonk, NY, USA) and Epitools (https://epitools.ausvet.com.au (accessed on 5 January 2024)) [51]. The level of significance was set to *p* < 0.05.

## 3. Results

This study encompasses 1582 sporting and utility trial dogs that have previously been described demographically in another peer-reviewed publication [47]. Here, the distribution of disciplines, sexual status, and lifetime age by breed and type of dog in the study group can be seen in Table 1. Of all dogs >1–2 years of age (*n* = 93), six (6.5%) had altered sexual status. In dogs 2–4 years old at the time of report, 55 (14.2%) were altered.

**Figure 1:**
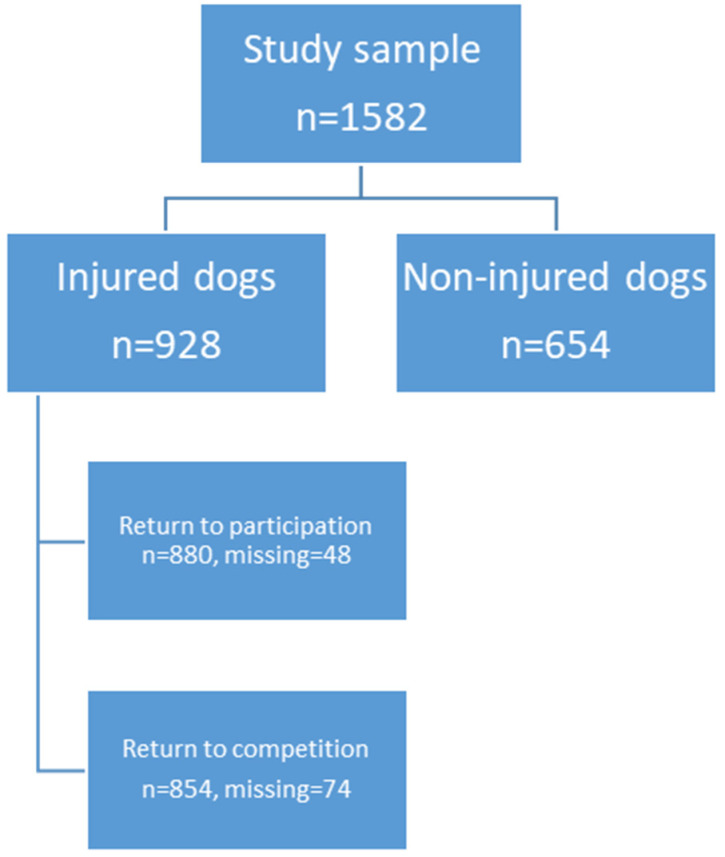
Flow chart illustrating the sporting and utility trial dogs included in this study.

Here, handlers of sporting and utility trial dogs reported that 928 (58.7%) dogs had experienced at least one injury and/or sign of injury across their lifetime to their current age, and the mean number of injuries was 1.6 per dog (Figure 1). In total, there were 1499 injuries reported in this study. Of the injured dogs, 403 (43.4%) had experienced one injury, 372 (40.1%) had experienced two to three injuries, and 116 (12.5%) had experienced four injuries or more. Concerning injury frequency, missing values occurred due to inconsistencies in the answers from some respondents. Following an audit of the injury data, thirty-seven dogs from the non-injury group were moved to the injury group since the respondents reported that the dogs had musculoskeletal conditions, e.g., osteoarthritis and spondylosis, which were defined as injuries in this study.

Amongst the injured dogs, the age category 4–6 years was the most common (*n* = 243, 26.2%) (Table 2). Of the injured dogs, *n* = 364 (39.2%) were intact females, *n* = 143 (15.4%) were altered females, *n* = 309 (33.3%) were intact males, and *n* = 112 (12.1%) altered males (Table 2). The median body weight of injured dogs was 24 kg (IQR 14 kg, *n* missing = 3), and of dogs reported without injury, it was 22 kg (IQR 15.5, *n* missing = 3). Handlers reported that nearly half of the dogs in the study (*n* = 715, 45%) had experienced an injury that kept the dog from participating in training or competition. Injury or signs of injury during or following participation in sport-specific training and competition, were reported in 394 (24.7%) and 109 (6.8%) of the dogs, respectively. In 534 (33.8%) of the dogs, the injury or signs of injury were perceived outside of sport-specific training or competition.

The respondents for the majority of the dogs in the injured group (*n* = 767, 83%) further specified the onset of injury complaints. The most common onset of injury appeared in the forest (*n* = 95,10.2%); because of slipping (*n* = 87,9.4%); during physical activity and for an unknown reason (*n* = 67, 7.2%); when crashing into something, e.g., the handler, a hurdle, or a decoy (*n* = 59, 6.4%); jumping (*n* = 49, 5.3%); playing with another dog (*n* = 49, 5.3%), and playing in other ways than with another dog (*n* = 49, 5.3%). Additional onsets of injury are presented in the Supplementary file, Table S2.

### 3.1. Distribution of Injuries According to Body Systems

The dogs had sustained injuries corresponding to eight various body systems according to the Pyramidion diagnosis system. Based on all injuries reported (*n* = 1499), muscular, joint, and dermatological injuries, along with unspecified injury complaints classified to the multiorgan system, were the most commonly reported. The distribution of injuries among dogs with at least one injury is presented in Table 3 and Table 4. Injuries are categorized by diagnoses and corresponding body systems according to the Pyramidion diagnosis system, and stratified by participation in various sporting and utility trial disciplines (Table 3). Muscular injuries were proportionally most common in dogs participating in messenger (*n* = 11, 33.3%), protection (*n* = 45, 26.6%), agility (*n* = 112, 26%), and search (*n* = 55, 24.3%) (Table 3). The highest proportion of joint and ligament injuries was observed in dogs participating in agility (*n* = 87, 20.2%) (Table 3).

Muscular and joint injuries were proportionally most common in Border Collies (*n* = 39, 29.3% and *n* = 35, 26.5%, respectively), while dermatological injuries were most common in Belgian Malinois and Labrador Retrievers (Table 4). In Table 4, the dogs are stratified and injury proportions are presented by the most common breeds and type represented in this study sample.

The most commonly reported injuries in the muscular system were muscle strain (*n* = 224) and degenerative tendinopathies (*n* = 63). In the dermatologic system, wound (*n* = 176) was most commonly reported, while in the multiorgan system it was lameness (*n* = 168), and in the joint and ligament system, traumatic ligament sprain (*n* = 90) was most frequent. In Table 5, the most common types of injury are presented by diagnoses and corresponding body systems.

### 3.2. Distribution of Injuries by Anatomical Body Locations

Based on all injuries reported (*n* = 1499), injuries to the thoracic, lumbar and lumbosacral spine, paw (including the pads, digits and nails), head, shoulder, and stifle were the most commonly reported (Supplementary file, Table S3).

Proportionally, thoracic, lumbar, and lumbosacral spine injuries were more common in dogs participating in protection; head injuries were more common in messenger dogs, paw injuries in area search dogs, shoulder injuries in area searching and tracking dogs, and stifle injuries in agility dogs. With regards to the distribution of injuries among the most represented breeds and type, back injuries were most common in mixed breeds, head, paw, and stifle injuries in Labrador Retrievers, and shoulder injuries in Border Collies. The distribution of injuries categorized by anatomical location and participation in various sporting and utility trial disciplines is presented in the Supplementary file, Table S3, and by the most commonly represented breeds and type in the Supplementary file, Table S4.

The most commonly reported injuries in the back were spondylosis and spinal dysfunction not otherwise specified. In the Supplementary file, Table S5, the most common types of injuries are presented by diagnoses and corresponding anatomical location.

### 3.3. Injury Proportions in Various Sporting and Utility Trial Disciplines, Breeds, Sex, Neuter Status, and Age

Wilson 95% confidence intervals and Chi-squared tests were used to describe frequencies and assess for differences in injury proportions in the independent variables.

When all dogs in the sample (*n* = 1582) were stratified according to participation in various disciplines, the highest injury proportion was observed amongst messenger dogs (*n* = 25, 75.8%). Dogs participating in agility, protection, and area search all showed injury proportions of 64% (Table 3). Participation in obedience was attributable to the lowest proportion (57.6%, CI (95%) 54.1–61.0), while rally obedience (58.4%, CI (95%) 54.4–62.3), agility (63.7%, CI (95%) 59.1–68.1), and utility trial (60.9%, CI (95%) 57.6–64.2) participation had higher injury proportions (Table 2). In the univariate analysis, there were differences in frequency distribution in dogs participating in agility (*p* = 0.013) and in utility trials (*p* = 0.050) (Table 2).

There were differences (*p* < 0.001) in frequency distribution among breeds with regard to being injured. Amongst the most common breeds in our sample, Belgian Malinois dogs had the highest injury proportion (72.9%, CI (95%) 64–80.4%), followed by Border Collies (71.4%, CI (95%) 63.2–78.4), and mixed-breeds (69.6%, CI (95%) 55.2–80.9) (Table 2 and Table 4). The lowest injury proportion was reported in Australian Shepherds (50%, CI (95%) 39.7–60.3%) (Table 2). The non-overlapping 95% confidence intervals of the Belgian Malinois and Border Collies, compared to those of the Australian Shepherds, German Shepherds, Shetland Sheepdogs, and the group of other purebred dogs, indicated a difference between these breeds and this group specifically (Table 2).

The frequency distribution in dogs that had been reported with any injury history varied by sex (*p* = 0.048) and neuter status (*p* < 0.001). The variation in sexual status was attributable to the highest injury proportion among males (61.5%, CI (95%) 57.8–65.0). We also observed a higher injury proportion among altered dogs (68.5%, CI (95%) 63.7–73.1), compared to intact dogs (Table 2).

Dogs with a higher lifetime age at the point of entering this study had larger injury proportions (*p* < 0.001), where the highest injury proportion was observed among dogs >10 years (79.8%, CI (95%) 70.6–86.7) and the lowest proportion among dogs <1–2 years of age (26.9%, CI (95%) 18.9–36.7) (Table 2).

### 3.4. Associations between Independent Variables and Injury History

Multivariate logistic regression analysis was used to analyze the relationship between participation in various disciplines, i.e., obedience, rally obedience, agility, utility trials, breeds, sex, neuter status, and lifetime age at the time of the report. We found that holding all other variables constant, the odds of injury history are 1.9 times higher in Border Collies (*p* = 0.003, adjusted OR 1.93, CI (95%) 1.25–2.99) and 2.5 times higher in Belgian Malinois dogs (*p* < 0.001, adjusted OR 2.51, CI (95%) 1.54–4.08 (Table 6). The higher the age of the dogs at the time of the report, the higher the odds of injury history, indicating dogs with higher lifetime age had more years to accumulate a history of injury (Table 6). Dogs 2–4 years had 1.8 times higher adjusted odds of being reported with injury, compared to dogs >10 years old which had almost 10 times the odds of any injury at that age (Table 6).

### 3.5. Return to Participation, Competition, and Performance

Due to the reported injury, rehabilitation interventions were provided to 591 (65.7%, missing *n* = 29) of the dogs in the injury group. Approximately four out of five (*n*= 759, 81.8%, missing value *n* = 48) injured dogs returned to participation in sport-specific training and three out of four (*n* = 690, 74.4%, missing value *n* = 74) dogs returned to competition. Among the injured dogs, 41 (4.4%) were not able to return to participation and 47 (5.1%) were not able to return to competition in their previous discipline. In 117 (12.6%) of the cases, the handler chose to dismiss their dog from competition in the previous discipline. Following injury, 129 (13.9%) performed at a higher level, 35 (3.8%) at a lower level, and 526 (56.7%) at the same level of competition.

With regards to the educational background of the rehabilitation providers, 279 (29.7%) of the handlers of dogs in the injury group consulted a registered animal health care professional, i.e., a registered physiotherapist with continuing education in veterinary medicine and rehabilitation, veterinarian, or veterinary nurse with continuing education in physical rehabilitation. A third (*n* = 325, 35%) were provided services by a non-registered rehabilitation practitioner, and a few (*n* = 16, 1.7%) by the dog handler. In 36 (3.9%) of the injured dogs, the respondent did not know the educational background of the rehabilitation provider.

### 3.6. Sensitivity Analyses

Excluding injury complaints that were not confirmed by a veterinarian decreased the frequencies and proportions reported in the analysis of involved body systems and anatomical body locations. The number of injured dogs decreased to 596 (37.7%) and the number of reported injuries decreased to 871 (58.1%). We observed that the proportions of reported muscular, dermatological, and unspecified pain conditions decreased when self-diagnosed injuries were excluded from the analysis (Table 3, Table 4 and Table S6A,B). More specifically, the proportions of distal limb injuries and unspecified muscular, pain, and soft tissue injuries, e.g., injuries to the skin, were lower in dogs with veterinary-confirmed diagnoses (Supplementary File, Tables S4 and S7A,B). The results from the sensitivity analyses are further presented in Supplementary File, Tables S6A,B and S7A,B.

## 4. Discussion

This descriptive and correlational study with cross-sectional design provides detailed insights regarding frequencies and types of lifetime injuries experienced amongst Swedish sporting and utility trial dogs and identifies important associations between breeds and injury history.

As hypothesized, there was an association where Border Collies, and also Belgian Malinois dogs, had higher adjusted odds of injury. We were not surprised to confirm that our hypothesis that higher lifetime age of the dogs would be associated with injury was true. A straightforward explanation for that finding is that older dogs have more years to accumulate injuries. Contrary to our hypothesis, neither participation in obedience, rally obedience, agility or utility trials, nor altered sexual status were associated with an injury history. As expected, dogs’ sex was not associated with higher odds of injury.

### 4.1. Anatomical Injury Locations

The results from our sample suggest that musculoskeletal injuries were common among competitive dogs. Injuries located on the thoracic, lumbar, and lumbosacral spine, shoulder, i.e., muscle strain, ligament sprain, and tendinopathies, and stifle, i.e., ligament injuries and patella luxation, were most common in the present study. Together with injuries to the head, i.e., tooth fractures and traumatic eye injuries, and distal limb, i.e., wounds, fractures, ligament sprains, and osteoarthritis, these anatomical locations are in line with previous studies in agility dogs [24,32]. Interestingly, we observed that the reported anatomical body locations of the two breeds with the highest injury proportions, Belgian Malinois dogs and Border Collies, are in line with the breed profiles based on veterinary care events registered within a pet insurance company [52].

Despite the evidence of the high physical demands dogs are subjected to during bite tasks in protection work [8], the results in our study indicate that neck injuries are uncommonly reported and the injury proportion is remarkably low compared to North American samples of agility dogs [32]. The proportions between countries and geographic regions already differed, according to a previous study, which supports our findings [32]. According to the respondents in a qualitative study published by our research group, several factors, e.g., protection work, the interaction between the dog and the handler, and the safety strategies incorporated by the handler, may serve as barriers or facilitators to canine health promotion. The protection dogs in our sample of Swedish utility trial dogs include dogs competing in several bite work classes. The Swedish Schutzhund class encompasses muzzle tasks, which have been argued as potentially harmful since the dogs stop the helper by long and short attacks while wearing a muzzle. In practice, this means that the dog collides with the head and muzzle into the helper. Despite the biomechanical forces acting upon the dogs during these challenges, the results from our study do not indicate that concussion or neck injuries are particularly common. However, concerning the thoracic, lumbar, and lumbosacral spine injuries, dogs participating in protection had slightly higher injury proportions. We also observed various proportions of thoracic, lumbar, and lumbosacral spine injuries between the breeds, with the highest injury proportion in mixed-breed dogs. According to the Swedish Working Dog Association national regulations [2], mixed-breed dogs are not allowed to compete in utility trial classes, except for Internationale Gebrauchshund Pruefung Rettungshunde.

### 4.2. Return to Participation, Competition, and Performance

Epidemiological studies addressing the return to competition in canine athletes are scarce and information is still mostly anecdotal. Compared to competitive agility dogs with stifle injury and subsequent surgery [39], a higher proportion of dogs returned to sport-specific participation and competition in our study. This was expected since various types of injuries were included in our study. In addition, an important proportion of injuries reported in our sample of dogs were minor, e.g., superficial skin wounds and broken nails, and according to sensitivity analyses, these injuries did not receive medical attention other than from the dog handler. Stifle injuries are considered more severe and with worse prognosis for return to competition [38]. Spinella et al. [31] recently described that in a large proportion of reported injury complaints in their sample of active working dogs, the signs of injury never recurred. The proportions of dogs returning to participation, competition, or level of performance were not specifically described in their study sample, which otherwise seemed similar to ours.

### 4.3. Injury Frequencies and Proportion

In agreement with another study in Scandinavian agility dogs [32], our survey included sporting and utility trial dogs obtaining any injury, not only injuries directly appearing during sports activity, and dog handlers’ self-diagnosed injuries over the lifetime of their dog. This may lead to higher injury proportions compared to studies targeting only sports-related injuries appearing directly during sports activity [24].

With regard to the dog handlers independently self-diagnosing injuries in their dogs, our results are in line with proportions reported by Spinella et al. [31]. In about one-fifth of the cases, the dog handler managed the injury without medical attention from a veterinarian. According to the sensitivity analysis conducted in our study, the dog handlers mainly managed muscular and dermatological injuries through self-diagnosis and self-care. Unspecified pain conditions, together with muscular and dermatological injuries, were perceived in a remarkably large proportion of the dogs without having a veterinary pathoanatomical diagnosis reported by the dog handler. In our sample, 38% of the dogs had pathoanatomical diagnoses confirmed by a veterinarian. One previous study in a Finnish sample [24] reported a similar proportion, whereas in North American samples [33,35], the proportion of dogs that sought medical attention from a veterinarian was higher. The characteristics of the injuries managed by the dog handlers themselves indicate that some are minor injuries heal well from the attention and care by the dog handler. The reason for not seeking medical attention for sporting and utility dogs with unspecified pain conditions is unknown. It is the authors’ experience that subtle signs of pain are challenging to interpret. Further, they may potentially go unnoticed by the dog handler in high-drive dogs [53]. In our study, it appeared that dog handlers noticed and reported signs of pain without taking their dog to a veterinarian. The dogs were possibly provided regular assessments and re-evaluations by animal health care professionals other than veterinarians, as previously described in agility dogs [24,27]. Other explanations why handlers do not consult a veterinarian with their dog may be that they have self-efficacy or are experienced enough to provide self-care to their dog suffering from a minor injury, or the financial costs related to veterinary consultations may cause the owner to delay veterinary consultation.

### 4.4. Onset of Injury

Injuries in our sample sometimes appeared alongside sport-specific training or competition. Still, a fourth of the dogs were injured during or following sport-specific training, and a lower proportion of dogs appeared injured during or following competition or trial. We have previously reported on dog handlers’ beliefs with regard to competitive tasks [29]. Tasks that were considered barriers to canine health by the dog handlers’ and potentially harmful for the dogs were crawling, staying in a group with other dogs, protection work, high ladder, heavy retrieving, jumping obstacles, and gun shots. Interestingly, the most commonly reported settings at the onset of injury were activity in the forest, injury appearing as a result of slipping, or during play with another dog or another way of playing, e.g., with a human. Regardless, slightly more than a fourth of the dogs sustained an injury in conjunction with sport-specific training and/or competition, which highlights the importance of an in-depth anamnesis regarding which other physically demanding activities sporting and utility trial dogs are executing in addition to their competitive discipline. As previously reported, many competitive dogs are active in several disciplines and may as well execute activities such as, e.g., canicross, bikejoring, or skijoring, or perform as herding or hunting dogs [47].

No matter if dogs are being kept as companion pets, or are being trained towards or already are participating in sports or utility trials, dog owners and handlers need to account for all types of physical activities in order to implement and facilitate functional recovery in their dogs. The injuries reported here were defined as injuries regardless of being an acute or acute-on-chronic injury, and therefore other activities than sport-specific training or competition might have caused both instant major acute trauma and minor repetitive tissue trauma over time in this sample of sporting and utility dogs. It was not within the scope of this study to assess for differences in injury proportions and relationships between competitive and companion dogs, e.g., of the same breed.

Therefore, the authors want to highlight that there are several reported onsets of injury that are situations for organizers of canine sporting events and utility trials to consider as potential triggers to injury. Onsets of injury appearing in the forest and slipping, possibly as a result of a combination of high speed and reduced surface friction, might be prevented to a higher extent when the causes are brought to the attention of the responsible organizers.

### 4.5. Associations between Injury History and Discipline, Breed, Sexual Status, and Age

To the authors’ knowledge, this study is the first to assess factors associated with injuries in competitive utility trial dogs. Since we have observed that Swedish sporting and utility trial dogs often participate in several disciplines, and according to our experience, some disciplines encompass similar tasks, e.g., Swedish Schutzhund involves tracking and searching tasks in the superior class, we were aware of the importance of capturing a broad sample. The prior literature on agility dogs indicated that there was no association between participation in several disciplines and injury appearing during training or competition [43]. We have previously reported that about half of the dogs in our sample concurrently participate in several competitive disciplines [47]. In this study, we estimated the potential relationships between injury history and participation in obedience, rally obedience, agility, and several utility disciplines collapsed into one. In order to obtain meaningful results with regard to the exposure from disciplines, we added them separately to a multivariate model. Controlling for all exposure variables, the findings indicate that there were no relationships between injury history and participation in any of the disciplines investigated. The explanation for this can be that dogs in our sample participated in several disciplines and were active in other physically demanding activities [47]. Unfortunately, we could not control for the latter since all physical activity data were obtained at the same point in time, i.e., cross-sectional, and we cannot know from this study if combinations of participation in disciplines or certain physical activity patterns antedate a history of injury.

In line with previous studies [32,40,41,54], we first confirmed higher injury proportions among Border Collies and Belgian Malinois dogs among the most common individual breeds and type of the dogs in our sample. Following adjusting for the exposure from other variables, Border Collies and Belgian Malinois dogs were associated with higher odds of injury history. These breeds being common in agility, obedience, and utility trial disciplines may lead to confounding effects on the results reported here. Nevertheless, another explanation for our findings is the combination of traits found in these breeds, e.g., boldness, intensity, speed, and task orientation, characteristics that may cause these dogs to expose themselves to and take greater risks during sport-specific activities and other physical activities.

In a previous study published by our research group, we observed that in contrast to studies on flyball and agility dogs, 77% of the dogs in our sample were sexually intact. The numbers are close to the opposite compared to the American samples [32,49]. Intact sexual status serving as a protective or risk factor has been elaborated on in other studies [34,44,45]. Potentially, the removal of the gonads and disruption of gonadal hormones is responsible for biological changes. In human athletes, there is a growing body of knowledge regarding the important role of female hormones on biological changes, athletic achievement, and injury risks [55]. The risk of certain injuries, e.g., anterior cruciate ligament tear, has been correlated with phases of the menstrual cycle and corresponding hormonal milieus [56]. Before controlling for the other exposure variables, we observed that altered dogs had a higher injury proportion compared to intact dogs in our sample. An important finding was that according to the multivariate analysis controlling for the effects of all independent variables, altered sexual status did not fall out as a risk factor for injury per the definition used in this study. In the sensitivity analyses, we found that a considerable proportion of the self-diagnosed injuries were minor, e.g., skin wounds and torn nails. The minor injuries are, according to the authors, less likely to be affected by biological changes related to altered sexual status, and they may potentially have influenced our results about neuter status. Research providing further insights on the relationships between various factors, e.g., nutrition, disruption of the canine hormone cycles, and injuries in sporting and utility dogs, are therefore still needed.

Our multivariate analysis captured that dogs at higher age at the time of reporting to this study had more years to accumulate a history of injury. As expected, the increasing injury proportion with higher age was significantly associated with any injury history sustained by the dog. This finding corresponds well with the results from other studies in agility dogs [28,34,40,43], and working utility dogs [41]. Due to the method of data collection used in this study, we do not know whether older dogs are actually more injury prone than younger dogs. Older dogs accumulate a history of injury during their lifetime and longitudinal epidemiological studies are needed to explore the incidence of injuries.

### 4.6. Strengths and Limitations

A strength of this study is that we targeted a broad distribution of injuries in sporting and utility trial dogs, from any injury complaint based on self-diagnosis to injuries that lead to medical attention confirmed by a veterinarian. We conducted sensitivity analyses to strengthen the internal validity of this study, and as a result, this study is reporting on any complaints in the full cross-sectional cohort and cases with medical attention from the sensitivity analyses in the Supplementary file.

We have also described time loss in injured dogs as the dogs being unable to participate in sport-specific training due to injury. In addition, we have described the return to participation, competition, and performance from dog handler-reported data [57,58].

In line with Bahr et al., we defined injury as tissue damage or other derangement of normal physical function, regardless of the presentation being sudden or gradual, from an acute or acute-on-chronic mode of onset [57]. We chose this definition regarding mode of onset since our experience is that many degenerative musculoskeletal conditions appear with a sudden onset to the dog handler, although all injuries are not acute. Instead, injuries sometimes present with a sudden onset, or from a repetitive etiology.

Another strength is that our survey approach made it possible to reach out to dogs participating in several sporting and utility trial disciplines covered by the main organization, the Swedish Kennel Club. In this way, we obtained a large amount of data, collected over a specific period.

Our results provide valuable insights on frequencies and types of injuries and musculoskeletal conditions in Swedish sporting and utility dogs, and our findings contribute to theories on how to further develop injury prevention strategies involving stakeholders, i.e., dog handlers, trainers, breeders, judges, organizers of competitions, animal health care professionals. Canine welfare may be facilitated by continuously refining the guidelines and regulations, and continuing to provide dog handler education, e.g., on breeding and various aspects of canine health and soundness implied from the findings in this study.

The limitations of this study are that participation was anonymous and it did not collect any demographic information about the dog handlers, or about their experience as dog handlers or dog trainers. Further, with self-reported data, there is a risk of sampling, confirmation, and recall bias, which are well-known challenges in questionnaire surveys [59,60]. In this study, we observed the presence of lifetime injuries, hence the respondents did not provide answers to at which age the injuries appeared.

The authors want to emphasize that no hypothesis testing was performed between disciplines or breeds and type of dog, and anatomical locations or body systems of the injuries reported here. A reason for this is the low numbers in the contingency tables for some of the anatomical body locations and body systems. A larger sample representing each of the disciplines and breeds is required to estimate differences or associations related to disciplines or breeds and injury locations. On the other hand, we have reported differences and associations between self-reported injury history and several exposure variables that antedated injury history.

Physical activity patterns have previously been reported in the same sample. It would have been interesting to explore associations between physical activity patterns and injury history. Unfortunately, such analyses were not feasible with this cross-sectional study design since it would not be possible to conclude whether the injury outcome preceded or was caused by physical activity characteristics. The design of this study, and several other epidemiological cross-sectional studies in sporting dogs, entails limitations when it comes to retrospective analysis of the risk of and protective factors to injury. The authors needed to aim for characteristics that antedated the outcome of injury history. The findings should be interpreted with caution, given that several exposures were not accounted for. Thus, the authors want to highlight the need for longitudinal epidemiological studies in canine sports and performance medicine, e.g., to appropriately assess for differences in injury proportions amongst competitive and companion dogs of the same breed.

## 5. Conclusions

In conclusion, we observed differences in reported injury proportion with regard to participation in agility and utility trials, breeds and type, sex, altered sexual status, and dogs’ age at the time of entering the study. Controlling for all exposure variables in our model, associations between injury history and Border Collies and Belgian Shepherds, and dogs with increasing lifetime age remained and indicated higher odds of self-reported injury history. Four out of five injured dogs returned to participation in sport-specific training and three out of four returned to competition. Estimations of injury proportions were sensitive to the injury complaint being self-diagnosed or confirmed by a veterinarian. Our results provide valuable insights on frequencies and types of injuries in sporting and utility dogs and contribute to theories on how to further develop injury prevention strategies involving, e.g., management routines and breeding, that potentially enhance canine welfare.

## Figures and Tables

**Table 1:** Demographics and characteristics of sporting and utility dogs (*n* = 1582). Data are presented as frequencies and proportions (%).

	Full Cohort	Australian Kelpie	Australian Shepherd	Border Collie	Belgian Malinois	German Shepherd Dog	Labrador Retriever	Shetland Sheepdog	Other Purebreds	Mixed breeds
*n* (%) of all dogs	1582	75 (4.7)	86 (5.4)	133 (8.4)	111 (7.0)	205 (13.0)	67 (4.2)	67 (4.2)	791 (50.0)	46 (2.9)
Discipline										
Obedience	790 (49.9)	45 (60.0)	61 (70.9)	83 (62.4)	47 (42.3)	86 (42.0)	40 (59.7)	11 (16.4)	525 (66.4)	14 (30.4)
Rally obedience	596 (37.7)	30 (40.0)	45 (52.3)	38 (28.6)	9 (8.1)	21 (10.2)	26 (38.8)	33 (49.3)	368 (46.5)	26 (56.5)
Agility	430 (27.2)	25 (33.3)	20 (23.3)	72 (54.1)	8 (7.2)	1 (0.5)	4 (6.0)	56 (83.6)	217 (27.4)	27 (58.7)
Utility trials *	847 (53.5)	49 (65.3)	55 (64.0)	44 (33.1)	99 (89.2)	194 (94.6)	41 (61.2)	3 (4.5)	361 (45.6)	1 (2.2)
Sexual status										
Intact female	692 (43.7)	37 (49.3)	34 (39.5)	62 (46.6)	45 (40.5)	93 (45.4)	19 (28.4)	31 (46.3)	353 (44.6)	18 (39.1)
Intact male	518 (32.7)	17 (22.7)	37 (43.0)	39 (29.3)	41 (36.9)	72 (35.1)	31 (46.3)	22 (32.8)	254 (32.1)	5 (10.9)
Spayed female	205 (13)	15 (20.0)	7 (8.1)	14 (10.5)	17 (15.3)	31 (15.1)	6 (9.0)	9 (13.4)	98 (12.4)	8 (17.4)
Neutered male	167 (10.6)	7 (9.3)	8 (9.3)	18 (13.5)	8 (7.2)	9 (4.4)	11 (16.4)	5 (7.5)	86 (10.9)	15 (32.6)
Age at report										
<1–2 years	93 (5.9)	3 (4.0)	11 (12.8)	11 (8.3)	8 (7.2)	7 (3.4)	1 (1.5)	8 (11.9)	43 (5.4)	1 (2.2)
2–4 years	387 (24.5)	21 (28.0)	14 (16.3)	22 (16.5)	39 (35.1)	55 (26.8)	20 (29.9)	18 (26.9)	186 (23.5)	12 (26.1)
4–6 years	428 (27.1)	17 (22.7)	24 (27.9)	42 (31.6)	19 (17.1)	59 (28.8)	17 (25.4)	17 (25.4)	222 (28.1)	11 (23.9)
6–8 years	268 (16.9)	12 (16.0)	20 (23.3)	25 (18.8)	19 (17.1)	34 (16.6)	13 (19.4)	11 (16.4)	130 (16.4)	4 (8.7)
8–10 years	207 (13.1)	12 (16.0)	12 (14.0)	19(14.3)	13 (11.7)	25 (12.2)	11 (16.4)	9 (13.4)	97 (12.3)	9 (19.6)
>10 years	94 (5.9)	6 (8.0)	5 (5.8)	10 (7.5)	2 (1.8)	7 (3.4)	3 (4.5)	3 (4.5)	53 (6.7)	5 (10.9)
Deceased	105 (6.6)	5 (6.7)	0 (0)	4 (3.0)	11 (9.9)	18 (8.8)	2 (3.0)	1 (1.5)	60 (7.6)	4 (8.7)

* Utility trial disciplines were defined as Swedish Schutzhund, tracking (SWDA), search (SWDA), messenger (SWDA), patrol (SWDA), International Utility Dog trials (tracking, obedience, protection, search and rescue), International Nordic Style, BH/VT, and mondioring, and were collapsed to one category. SWDA = Swedish Working Dog Association.

**Table 2:** Number and proportion (%) of injured dogs (*n* = 1582), by participation in various disciplines, breeds or type of dog, age categories at the time of report categories, and sexual status. Estimated Wilson confidence intervals (95%) of the injury proportions and *p*-values from asymptotic significance Chi-squared tests (2-sided) are presented.

	*n* of Dogs	*n* (%) of Injured Dogs	95% CI of the Injury Proportions	*p*-Value
Disciplines *				
Obedience	790	455 (57.6)	54.1–61.0	0.102
Rally obedience	596	348 (58.4)	54.4–62.3	0.865
Agility	430	274 (63.7)	59.1–68.1	0.013
Utility trials	847	516 (60.9)	57.6–64.2	0.050
Breed or type of dog				<0.001
Australian Kelpie	76	40 (52.6)	41.6–63.5	
Australian Shepherd	86	43 (50)	39.7–60.3	
Border Collie	133	95 (71.4)	63.2–78.4	
Belgian Malinois	111	81 (73)	64.1–80.4	
German Shepherd Dog	205	115 (56.1)	49.3–62.7	
Labrador Retriever	67	38 (56.7)	44.8–67.9	
Shetland Sheepdog	67	34 (50.7)	39.1–62.4	
Mixed breeds	46	32 (69.6)	55.2–80.9	
Other purebreds	791	450 (56.9)	53.4–60.3	
Sexual status				
Male	685	421 (61.5)	57.8–65.0	0.048
Neuter status				
Altered	372	255 (68.5)	63.7–73.1	<0.001
Age				<0.001
<1–2 years	93	25 (26.9)	18.9–36.7	
2–4 years	387	162 (41.9)	37.1–46.8	
4–6 years	428	243 (56.8)	52–61.4	
6–8 years	268	187 (69.8)	64.0–75.0	
8–10 years	207	158 (76.3)	70.1–81.6	
>10 years	94	75 (79.8)	70.6–86.7	
Deceased	105	78 (74.3)	65.2–81.7	

* Dogs were stratified by participation in various disciplines; agility, obedience, rally obedience, or any of the utility trial disciplines. Utility trial disciplines were defined as Swedish Schutzhund, tracking (SWDA), search (SWDA), messenger (SWDA), patrol (SWDA), International Utility Dog trials (tracking, obedience, protection, search and rescue), International Nordic Style, BH/VT, and mondioring, and were collapsed to one category. SWDA = Swedish Working Dog Association. CI = confidence intervals.

**Table 3:** Injured body system, by disciplines, in a sample of Swedish sporting and utility trial dogs (*n* = 1582).

	Full Cohort	Obedience	Rally Obedience	Agility	Utility Trials *	Protection	Tracking	Search	Messenger
N of injured dogs	928 (58.7)	455 (57.6)	348 (58.4)	274 (63.7)	516 (60.9)	108 (63.9)	403 (60.4)	144 (63.7)	25 (75.8)
Pyramidion diagnosis system	*n* (%)	*n* (%)	*n* (%)	*n* (%)	*n* (%)	*n* (%)	*n* (%)	*n* (%)	*n* (%)
Muscular	330 (20.9)	169 (21.4)	125 (20.0)	112 (26.0)	189 (22.3)	45 (26.6)	141 (21.1)	55 (24.3)	11 (33.3)
Joint and ligament	234 (14.8)	114 (14.4)	88(14.8)	87 (20.2)	123 (14.5)	26 (15.4)	94 (14.1)	34 (15.0)	5 (15.2)
Skeletal	144 (9.1)	85 (10.8)	57 (9.6)	29 (6.7)	95 (11.2)	20 (11.8)	82 (12.3)	22 (9.7)	4 (12.1)
Dermatologic	271 (17.1)	131 (16.6)	104 (17.4)	65 (15.1)	155 (18.3)	35 (20.7)	123 (18.4)	53 (23.5)	6 (18.2)
Multiorgan	274 (17.3)	133 (16.8)	119 (20.0)	82 (19.1)	142 (16.8)	24 (14.2)	107 (16.0)	45 (19.9)	6 (18.2)
Ophthalmologic	17 (1.1)	6 (0.8)	6 (1.0)	2 (0.5)	9 (1.1)	1 (0.6)	7 (1.0)	2 (0.9)	1 (3.0)
Nervous	6 (0.4)	4 (0.5)	5 (0.8)	0 (0.0)	4 (0.5)	0 (0.0)	4 (0.6)	0 (0.0)	0 (0.0)
Digestion	44 (2.8)	29 (3.7)	17 (2.9)	7 (1.6)	28 (3.3)	7 (4.1)	24 (3.6)	9 (4.0)	2 (6.1)

* Utility trial disciplines were defined as Swedish Schutzhund, tracking (SWDA), search (SWDA), messenger (SWDA), patrol (SWDA), International Utility Dog trials (tracking, obedience, protection, search and rescue), International Nordic Style, BH/VT, and mondioring, and were collapsed to one category. SWDA = Swedish Working Dog Association.

**Table 4:** Injured body system, by breeds and type, in a sample of Swedish sporting and utility trial dogs (*n* = 1582). Only the most represented breeds (≥50 dogs per breed) are individually reported.

	Full Cohort	Australian Kelpie	Australian Shepherd	Border Collie	Belgian Malinois	German Shepherd	Labrador Retriever	Shetland Sheepdog	Other Purebreds	Mixed Breeds
N of injured dogs	928 (58.7)	40 (52.6)	43 (50)	95 (71.4)	81 (72.9)	115 (56.1)	38 (56.7)	34 (50.7)	450 (56.9)	32 (69.6)
Pyramidion diagnosis system	*n* (%)	*n* (%)	*n* (%)	*n* (%)	*n* (%)	*n* (%)	*n* (%)	*n* (%)	*n* (%)	*n* (%)
Muscular	330 (20.9)	15 (19.7)	21 (24.4)	39 (29.3)	23 (20.7)	43 (21.0)	11 (16.4)	16 (23.9)	152 (19.2)	10 (21.4)
Joint and ligament	234 (14.8)	9 (11.8)	11 (12.8)	35 (26.5)	19 (17.1)	27 (13.2)	10 (14.9)	11 (16.4)	103 (13.0)	9 (19.6)
Skeletal	144 (9.1)	4 (5.3)	9 (10.5)	7 (5.3)	10 (9.0)	20 (9.8)	6 (9.0)	0 (0.0)	85 (10.7)	3 (6.5)
Dermatologic	271 (17.1)	9 (11.8)	12 (14.0)	24 (18.0)	32 (28.8)	27 (13.2)	19 (28.4)	1 (1.5)	139 (17.6)	8 (17.4)
Multiorgan	274 (17.3)	15 (19.7)	9 (10.5)	32 (24.1)	26 (23.4)	31 (15.1)	10 (14.9)	9 (13.4)	135 (17.1)	7 (15.2)
Ophthalmologic	17 (1.1)	0 (0.0)	0 (0.0)	0 (0.0)	1 (0.9)	1 (0.5)	4 (6.0)	0 (0.0)	11 (1.4)	0 (0.0)
Nervous	6 (0.4)	1 (1.3)	1 (1.2)	0 (0.0)	1 (0.9)	0 (0.0)	0 (0.0)	0 (0.0)	1 (0.1)	2 (4.3)
Digestion	44 (2.8)	1 (1.3)	6 (7.0)	4 (3.0)	7 (6.3)	6 (2.9)	4 (6.0)	2 (3.0)	13 (1.6)	1 (2.2)

**Table 5:** The most commonly reported injuries. Injuries are categorized by diagnosis and corresponding body system according to the Pyramidion diagnosis system. Data are presented in frequencies.

Pyramidion Diagnosis System	Most Common	Second Most Common	Third Most Common	Fourth Most Common
**Muscular**	Muscle strain. (*n* = 224)	Metabolic, nutritional, degenerative/dystrophic changes. Tendon, tendon sheath, bursa. (*n* = 63)	Muscle Pain. (*n* = 21)	Myositis. (*n* = 18)
**Joint and ligament**	Ligament sprain, traumatic. (*n* = 90)	Osteoarthritis. (*n* = 67)	Cruciate Ligament Rupture. (*n* = 23)	Osteochondrosis and/or osteochondrosis dissecans. (*n* = 20)
**Skeletal**	Fracture. (*n* = 60)	Spondylosis. (*n* = 47)	Herniated disc. (*n* = 28)	Lumbosacral syndrome. (*n* = 20)
**Dermatologic**	Wound. *(n* = 176)	Torn nail. (*n* = 117)	Puncture wound. (*n* = 25)	Signs of injury or disease N.O.S. (*n* = 2)
**Multiorgan**	Lameness. (*n* = 168)	Pain. (*n* = 44)	Spinal dysfunction N.O.S. (*n* = 41)	Traumatic injury. N.O.S. (*n* = 35)
**Ophthalmologic**	Traumatic injury, foreign body, dislocation, thermal injury. (*n* = 13)	Traumatic injury, foreign body, dislocation, thermal injury. (*n* = 5)	N.A.	N.A.
**Nervous**	Brain concussion. (*n* = 2)	Fibrocartilaginous embolism. (*n* = 2)	Inflammation, infection (*n* = 1), Traumatic injury. Peripheral nerves. (*n* = 1)	N.A.
**Digestion**	Traumatic injury. (Tooth fracture). (*n* = 41)	Traumatic injury. Oral cavity, throat, esophagus. (*n* = 4)	N.A.	N.A.

N.O.S. = Not Otherwise Specified; N.A. = Not applicable.

**Table 6:** Results from multivariate logistic regression analysis estimating the relationships between independent variables with the binary outcome variable, i.e., any injury history, in the full cohort (*n* = 1582).

Independent Variable	B	S.E.	*p*-Value	OR	95% CI for OR	
					Lower	Upper
**Disciplines**						
Obedience	0.080	0.117	0.495	1.08	0.862	1.361
Rally obedience	0.038	0.127	0.765	1.04	0.809	1.333
Agility	−0.248	0.153	0.106	0.78	0.578	1.054
Utility trial	0.162	0.144	0.263	1.18	0.886	1.559
**Breeds and type**						
Other purebreds	REFERENCE		<0.001			
Mixed breed	0.424	0.351	0.228	1.53	0.767	3.042
Australian Shepherd	−0.245	0.258	0.343	0.78	0.472	1.298
Australian Kelpie	−0.271	0.247	0.273	0.76	0.470	1.239
Border Collie	0.659	0.222	0.003	1.93	1.250	2.990
Belgian Malinois	0.919	0.248	<0.001	2.51	1.543	4.078
German Shepherd Dog	−0.009	0.181	0.961	0.99	0.695	1.413
Labrador Retriever	−0.013	0.273	0.963	0.99	0.578	1.687
Shetland Sheepdog	−0.206	0.284	0.467	0.81	0.466	1.419
**Sex**						
Male	0.180	0.111	0.106	1.20	0.962	1.488
**Neuter status**						
Neutered/spayed	0.214	0.136	0.117	1.24	0.948	1.617
**Age at report**						
<1–2 years	REFERENCE		<0.001			
2–4 years	0.591	0.266	0.026	1.81	1.072	3.039
4–6 years	1.213	0.264	<0.001	3.36	2.004	5.647
6–8 years	1.749	0.282	<0.001	5.75	3.307	9.991
8–10 years	2.065	0.300	<0.001	7.88	4.382	14.177
<10 years	2.269	0.363	<0.001	9.67	4.746	19.682
Deceased	1.948	0.339	<0.001	7.02	3.614	13.624

Hosmer and Lemeshow test: Chi-squared 9.606, df 8, significance 0.294. CI = confidence intervals, OR = odds ratio, S.E. = standard error.

## Data Availability

The raw data supporting the conclusions of this article will be made available by the authors, upon reasonable request.

## Supplementary Materials

The following supporting information can be downloaded at: https://www.mdpi.com/article/10.3390/ani14030398/s1, Table S1. Items and variables regarding demographics and health history targeting characteristics of injury complaints, onset of injury, return to sport-specific activity and physical rehabilitation services in an online questionnaire survey targeting Swedish sporting and utility dogs (*n* = 1582). Table S2. Onset of injury complaints reported in a sample of Swedish sporting and utility trial dogs (*n* = 1582). There were 928 dogs in the injury group. Data are presented in frequencies and proportions (%). Table S3. Injured anatomical body location, by disciplines, in a sample of Swedish sporting and utility trial dogs (*n* = 1582). Table S4. Injured anatomical body location, by individual breeds and type, in a sample of Swedish sporting and utility trial dogs (*n* = 1582). Table S5. The most commonly reported anatomical body locations amongst all reported injury complaints (*n* = 1499), in sample of sporting and utility trial dogs (*n* = 1582). Table S6A. Injured body system, by disciplines, in a sample of Swedish sporting and utility trial dogs (*n* = 1582) following sensitivity analysis excluding self-diagnosed injuries. Table S6B. Injured body system, by individual breeds and type, in a sample of Swedish sporting and utility trial dogs (*n* = 1582) following a sensitivity analysis excluding self-diagnosed injuries. Table S7A. Injured anatomical location, by disciplines, in a sample of Swedish sporting and utility trial dogs (*n* = 1582) following sensitivity analysis excluding self-diagnosed injuries. Table S7B. Injured anatomical location, by individual breeds and type, in a sample of Swedish sporting and utility trial dogs (*n* = 1582) following sensitivity analysis excluding self-diagnosed injuries.
