# Supplementary material for: Injuries and Associated Factors in Swedish Sporting and Utility Trial Dogs—A Cross-Sectional Study
Source: Animals (Basel). 2024 Jan 26;14(3):398. doi: 10.3390/ani14030398 (PMC10854622; doi:10.3390/ani14030398)
Supplement: Supplementary file 1 [file animals-14-00398-s001.zip › animals-2773816-supplementary.pdf]

Supplementary File

Table S1. Items and variables regarding demographics and health history targeting characteristics of injury complaints, onset of injury, return to sport-specific activity and physical rehabilitation services in an online questionnaire survey targeting Swedish sporting and utility dogs (*n*=1582).

Topic	Variable	Categories
Demographics	Age	< 1 year/1-2 years/2-4 years/4-6 years/6-8 years/8-10 years/>10 years/Deceased
	Body weight	Kilograms
	Sexual status	Sexually intact male/Neutered male/Sexually intact female/Spayed female
	Breed	Breed by FCI/breed acknowledged by the Swedish Kennel Club/mixed breed
Health history	Injury frequency	Never/Once/2–3 times/4 times or more
	Self-diagnosis	Open ended. Classified according to a diagnostic coding system (Pyramidion) with a hierarchical structure
	Veterinarian confirmed diagnosis	yes/no
	Veterinary diagnosis	Open ended. Classified according to a diagnostic coding system (Pyramidion) with a hierarchical structure
	Injury location	Categories defined by predetermined body systems in Pyramidion diagnosis system
	Injury location	Categories defined according to anatomical body location described by the respondent
	Onset of injury complaint during training	yes/no
	Onset of injury complaint during competition	yes/no
	Onset of injury complaint	Categories defined according to situation described by the respondent.
	Level of severity (injury kept the dog from training or competition)	yes/no
	Return to sport-specific training	Chose not to let the dog go back/The dog was not able to go back/Dog got back and performed at a lower level/Dog got back and performed at the same level/Dog got back and performed at a higher level
	Return to competition	Chose not to let the dog go back/The dog was not able to go back/Dog got back and performed at a lower level/Dog got back and performed at the same level/Dog got back and performed at a higher level
	Rehabilitation services	yes/no
	Educational background of the rehabilitation provider	Animal health care professional (registered physiotherapist with continuing education in veterinary medicine and rehabilitation, registered veterinarian or veterinary nurse with continuing education in physical rehabilitation, /Other rehabilitation practitioner (e.g., veterinary technician with continuing education in physical rehabilitation, canineopath, massage therapist, chiropractor with continuing education in animal rehabilitation, naprapath with continuing education in animal rehabilitation, non-registered dog physiotherapist)/Dog handler/Do not know/Other

Table S2. Onset of injury complaints reported in a sample of Swedish sporting and utility trial dogs (*n*=1582). There were 928 dogs in the injury group. Data are presented in frequencies and proportions (%).

	n (%) of dogs
n of dogs, missing 161	767
Agility activity	43 (4.6)
Crashed	59 (6.4)
Dog fight	16 (1.7)
During physical activity and for unknown reason	67 (7.2)
Falling down	25 (2.7)
Flyball activity	1 (0.0)
Got cut or burned	43 (4.6)
Got stuck	47 (5.1)
Herding activity	8 (0.9)
Hunting activity	14 (1.5)
In the forest	95 (10.2)
Jumping	49 (5.3)
Other, e.g., unattended in the garden	14 (1.5)
Penetrated by sharp object	24 (2.6)
Playing other than with another dog	49 (5.3)
Playing with another dog	49 (5.3)
Protection activity	25 (2.7)
Retrieving activity	11 (1.2)
Reward situation	27 (2.9)
Slipped	87 (9.4)
Walked upon, e.g., by human or animal	14 (1.5)

Table S3. Injured anatomical body location, by disciplines, in a sample of Swedish sporting and utility trial dogs (*n*=1582).

	Full cohort	Obedience	Rally obedience	Agility	Utility (any)	Protection	Tracking	Search	Messenger
n of dogs participating	1582	790	596	430	847	169	667	226	33
n (%) of injured dogs	928 (58.7)	455 (57.6)	348 (58.4)	274 (63.7)	516 (60.9)	108 (63.9)	403 (60.4)	144 (63.7)	25 (75.8)
**Anatomical body location**	***n* (%)**	***n* (%)**	***n* (%)**	***n* (%)**	***n* (%)**	***n* (%)**	***n* (%)**	***n* (%)**	***n* (%)**
**Head**	64 (4.0)	36 (4.6)	25 (4.2)	9 (2.1)	40 (4.7)	10 (5.9)	33 (4.9)	12 (5.3)	4 (12.1)
**Neck**	13 (0.8)	6 (0.8)	4 (0.7)	3 (0.7)	8 (0.9)	1 (0.6)	5 (0.79	4 (1.8)	0 (0.0)
**Thoracic, lumbar, lumbosacral spine**	159 (10.1)	90 (11.4)	73 (12.2)	43 (10.0)	89 (10.5)	24 (14.2)	73 (10.9)	28 (12.4)	3 (9.1)
**Ribs or side**	1 (0.1)	0 (0.0)	0 (0.0)	0 (0.0)	1 (0.1)	1 (0.6)	1 (0.2)	0 (0.0)	0 (0.0)
**Pelvis**	3 (0.2)	1 (0.1)	1 (0.2)	0 (0.0)	3 (0.4)	0 (0.0)	3 (0.4)	0 (0.0)	1 (3.0)
**Tail**	15 (1.0)	9 (1.1)	5 (0.8)	3 (0.7)	8 (0.9)	1 (0.6)	6 (0.9)	1 (0.4)	1 (3.0)
**CNS**	4 (0.3)	2 (0.3)	3 (0.5)	0 (0.0)	3 (0.4)	0 (0.0)	3 (0.4)	0 (0.0)	0 (0.0)
**PNS**	2 (0.1)	2 (0.3)	2 (0.3)	0 (0.0)	1 (0.1)	0 (0.0)	1 (0.1)	0 (0.0)	0 (0.0)
**Shoulder**	63 (4.0)	39 (4.9)	27 (4.5)	20 (4.7)	40 (4.7)	7 (4.1)	36 (5.4)	13 (5.8)	1 (3.0)
**Brachium**	7 (0.4)	4 (0.5)	2 (0.3)	2 (0.5)	6 (0.7)	2 (1.2)	5 (0.7)	2 (0.9)	1 (3.0)
**Elbow**	15 (0.9)	6 (0.8)	7 (1.2)	3 (0.7)	8 (0.9)	2 (1.2)	5 (0.7)	0 (0.0)	1 (3.0)
**Antebrachium**	1 (0.1)	1 (0.1)	0 (0.0)	0 (0.0)	1 (0.1)	0 (0.0)	1 (0.2)	0 (0.0)	0 (0.0)
**Carpus**	30 (1.9)	17 (2.2)	7 (1.2)	6 (1.4)	24 (2.8)	7 (4.1)	16 (2.4)	11 (4.9)	2 (6.1)
**Forelimb N.O.S.**	48 (3.0)	28 (3.5)	27 (4.5)	14 (3.3)	27 (3.2)	4 (2.4)	20 (3.0)	3 (1.3)	1 (3.0)

**Groin**	13 (0.8)	9 (1.1)	4 (0.7)	7 (1.6)	6 (0.7)	0 (0.0)	6 (0.9)	0 (0.0)	1 (3.0)
**Hip**	21 (1.3)	8 (1.0)	4 (0.7)	8 (1.9)	11 (1.3)	1 (0.6)	8 (1.2)	3 (1.3)	1 (3.0)
**Thigh**	24 (1.5)	8 (1.0)	4 (0.7)	5 (1.2)	20 (2.4)	10 (5.9)	13 (1.9)	6 (2.7)	1 (3.0)
**Stifle**	69 (4.4)	33 (4.2)	32 (5.4)	29 (6.7)	32 (3.8)	2 (1.2)	28 (4.2)	5 (2.2)	2 (6.1)
**Tibial/fibular**	2 (0.1)	2 (0.3)	0 (0.0)	0 (0.0)	2 (0.2)	0 (0.0)	2 (0.3)	1 (0.4)	0 (0.0)
**Hock**	15 (0.9)	6 (0.8)	8 (1.3)	8 (1.9)	6 (0.7)	2 (1.2)	6 (0.9)	1 (0.4)	0 (0.0)
**Hindlimb N.O.S.**	50 (3.2)	23 (2.9)	27 (4.5)	21 (4.9)	17 (2.0)	3 (1.8)	11 (1.6)	5 (2.2)	1 (3.0)
**Paw**	63 (4.0)	29 (3.7)	34 (5.7)	14 (3.3)	28 (3.3)	4 (2.4)	23 (3.4)	13 (5.8)	0 (0.0)
**Pad**	62 (3.9)	31 (3.9)	16 (2.7)	13 (3.0)	40 (4.7)	9 (5.3)	32 (4.8)	9 (4.0)	2 (6.1)
**Digit**	120 (7.6)	66 (8.4)	50 (8.4)	41 (9.5)	70 (8.3)	11 (6.5)	60 (9.0)	21 (9.3)	2 (6.1)
**Nail**	114 (7.2)	51 (6.5)	45 (7.6)	30 (7.0)	61 (7.2)	13 (7.7)	45 (6.7)	21 (9.3)	2 (6.1)
**Limb N.O.S.**	33 (2.0)	17 (2.2)	10 (1.7)	8 (1.9)	17 (2.0)	4 (2.4)	13 (2.0)	9 (4.0)	0 (0.0)
**Bone N.O.S.**	3 (0.2)	1 (0.1)	0 (0.0)	0 (0.0)	3 (0.4)	0 (0.0)	3 (0.5)	1 (0.4)	0 (0.0)
**Joint N.O.S.**	52 (3.3)	23 (2.9)	16 (2.7)	15 (3.5)	34 (4.0)	10 (5.9)	23 (3.4)	8 (3.5)	0 (0.0)
**Muscle N.O.S.**	142 (9.0)	63 (8.0)	47 (7.9)	54 (12.6)	81 (9.6)	20(11.8)	57 (8.6)	25 (11.1)	4 (12.1)
**Pain N.O.S.**	146 (9.2)	74 (9.4)	58 (9.7)	45 (10.5)	74 (8.7)	8 (4.7)	56 (8.4)	26 (11.5)	4 (12.1)
**Soft tissue N.O.S.**	72 (4.6)	38 (4.8)	24 (4.0)	15 (3.5)	48 (5.7)	15 (8.9)	40 (6.0)	17 (7.5)	3 (9.1)
**Tendon N.O.S.**	8 (0.5)	4 (0.5)	4 (0.7)	0 (0.0)	6 (0.7)	2 (1.2)	6 (0.9)	3 (1.3)	0 (0.0)

N.O.S. = Not otherwise specified

Table S4. Injured anatomical body location, by individual breeds and type, in a sample of Swedish sporting and utility trial dogs (*n*=1582).

	**Full cohort**	**Australian Kelpie**	**Australian Shepherd**	**Border Collie**	**Belgian Malinois**	**German Shepherd Dog**	**Labrador Retriever**	**Shetland Sheepdog**	**Other Purebreed**	**Mixed Breed**
n of dogs participating	1582	76	86	133	111	205	67	67	791	46
n (%) of injured dogs	928 (58.7)	40 (52.6)	43 (50)	95 (71.4)	81 (72.9)	115 (56.1)	38 (56.7)	34 (50.7)	450 (56.9)	32 (69.6)
**Anatomical body location**	***n* (%)**	***n* (%)**	***n* (%)**	***n* (%)**	***n* (%)**	***n* (%)**	***n* (%)**	***n* (%)**	***n* (%)**	***n* (%)**
**Head**	64 (4.0)	1 (1.3)	6 (7.0)	4 (3.0)	8 (7.2)	9 (4.4)	8 (11.9)	2 (3.0)	25 (3.2)	1 (2.2)
**Neck**	13 (0.8)	0 (0.0)	0 (0.0)	0 (0.0)	2 (1.8)	1 (0.5)	0 (0.0)	0 (0.0)	10 (1.3)	0 (0.0)
**Thoracic, lumbar, lumbosacral spine**	159 (10.1)	5 (6.6)	9 (10.5)	10 (7.5)	12 (10.8)	24 (11.7)	5 (7.5)	4 (6.0)	81 (10.2)	9 (19.6)
**Ribs or side**	1 (0.1)	0 (0.0)	0 (0.0)	0 (0.0)	1 (0.9)	0 (0.0)	0 (0.0)	0 (0.0)	0 (0.0)	0 (0.0)
**Pelvis**	3 (0.2)	0 (0.0)	0 (0.0)	0 (0.0)	1 (0.9)	0 (0.0)	0 (0.0)	0 (0.0)	2 (0.3)	0 (0.0)
**Tail**	15 (0.9)	1 (1.3)	0 (0.0)	2 (1.5)	0 (0.0)	3 (1.5)	0 (0.0)	0 (0.0)	9 (1.1)	0 (0.0)
**CNS**	4 (0.3)	1 (1.3)	1 (1.2)	0 (0.0)	1 (0.9)	0 (0.0)	0 (0.0)	0 (0.0)	0 (0.0)	1 (2.2)
**PNS**	2 (0.1)	0 (0.0)	0 (0.0)	0 (0.0)	0 (0.0)	0 (0.0)	0 (0.0)	0 (0.0)	1 (0.1)	1 (2.2)
**Shoulder**	63 (4.0)	4 (5.3)	2 (2.3)	10 (7.5)	5 (4.5)	7 (3.4)	2 (3.0)	1 (1.5)	30 (3.8)	2 (4.3)
**Brachium**	7 (0.4)	0 (0.0)	0 (0.0)	1 (0.8)	1 (0.9)	2 (1.0)	0 (0.0)	0 (0.0)	3 (0.4)	0 (0.0)
**Elbow**	15 (0.9)	0 (0.0)	0 (0.0)	1 (0.8)	0 (0.0)	3 (1.5)	2 (3.0)	0 (0.0)	8 (1.0)	1 (2.2)
**Antebrachium**	1 (0.1)	0 (0.0)	0 (0.0)	0 (0.0	0 (0.0)	0 (0.0)	0 (0.0)	0 (0.0)	1 (0.1)	0 (0.0)
**Carpus**	30 (1.9)	3 (3.9)	1 (1.2)	3 (2.3)	5 (4.5)	5 (2.4)	0 (0.0)	1 (1.5)	11 (1.4)	1 (2.2)
**Forelimb**	48 (3.0)	4 (5.3)	3 (3.5)	3 (2.3)	3 (2.7)	6 (2.9)	2 (3.0)	1 (1.5)	24 (3.0)	2(4.4)
**Groin**	13 (0.8)	0 (0.0)	1 (1.2)	2 (1.5)	0 (0.0)	0 (0.0)	0 (0.0)	1 (1.5)	9 (1.1)	0 (0.0)

**Hip**	21 (1.3)	0 (0.0)	1 (1.2)	4 (3.0)	1 (0.9)	4 (2.0)	1 (1.5)	2 (3.0)	8 (1.0)	0 (0.0)
**Thigh**	24 (1.5)	1 (1.3)	2 (2.3)	1 (0.8)	3 (2.7)	9 (4.4)	0 (0.0)	0 (0.0)	7 (0.9)	1 (2.2)
**Stifle**	69 (4.4)	3 (3.9)	5 (5.8)	7 (5.3)	3 (2.7)	3 (1.5)	5 (7.5)	2 (3.0)	37 (4.7)	4 (8.7)
**Tibial/fibular**	2 (0.1)	0 (0.0)	0 (0.0)	0 (0.0)	0 (0.0)	0 (0.0)	0 (0.0)	0 (0.0)	2 (0.3)	0 (0.0)
**Hock**	15 (0.9)	0 (0.0)	0 (0.0)	1 (0.8)	3 (2.7)	1 (0.5)	0 (0.0)	6 (9.0)	4 (0.5)	0 (0.0)
**Hindlimb**	50 (3.2)	1 (1.3)	2 (2.3)	9 (6.8)	3 (2.7)	2 (1.0)	2 (3.0)	1 (1.5)	26 (3.3)	4 (8.7)
**Paw**	63 (4.0)	4 (5.3)	4 (4.7)	4 (3.0)	3 (2.7)	10 (4.9)	3 (4.5)	1 (1.5)	33 (4.2)	1 (2.2)
**Pad**	62 (3.9)	1 (1.3)	2 (2.3)	8 (6.0)	8 (7.2)	8 (3.9)	2 (3.0)	0 (0.0)	31 (3.9)	2 (4.3)
**Digit**	120 (7.6)	6 (7.9)	7 (8.1)	17 (12.8)	9 (8.1)	10 (4.9)	8 (11.9)	1 (1.5)	61 (7.7)	1 (2.2)
**Nail**	114 (7.2)	2 (2.6)	5 (5.8)	9 (6.8)	12 (10.8)	6 (2.9)	11 (16.4)	0 (0.0)	65 (8.2)	4 (8.7)
**Limb N.O.S.**	31 (2.0)	0 (0.0)	0 (0.0)	7 (5.3)	3 (2.7)	2 (1.0)	3 (4.5)	2 (3.0)	12 (1.5)	2 (4.3)
**Bone N.O.S.**	3 (0.2)	0 (0.0)	0 (0.0)	0 (0.0)	0 (0.0)	0 (0.0)	0 (0.0)	0 (0.0)	3 (0.4)	0 (0.0)
**Joint N.O.S.**	52 (3.3)	1 (1.3)	4 (4.7)	7 (5.3)	5 (4.5)	10 (4.9)	2 (3.0)	4 (6.0)	19 (2.4)	0 (0.0)
**Muscle N.O.S.**	142 (9.0)	10 (13.2)	12 (14.0)	18 (13.5)	11 (9.9)	18 (8.8)	7 (10.4)	5 (7.5)	57 (7.2)	4 (8.7)
**Pain N.O.S.**	146 (9.2)	9 (11.8)	5 (5.8)	13 (9.8)	12 (10.8)	14 (6.8)	4 (6.0)	5 (7.5)	82 (10.4)	2 (4.3)
**Soft tissue N.O.S.**	72 (4.6)	1 (1.3)	3 (3.5)	4 (3.0)	13 (11.7)	8 (3.9)	7 (10.4)	1 (1.5)	33 (4.2)	2 (4.3)
**Tendon N.O.S.**	8 (0.5)	0 (0.0)	0 (0.0)	1 (0.8)	1 (0.9)	1 (0.5)	0 (0.0)	0 (0.0)	5 (0.6)	0 (0.0)

N.O.S. = Not otherwise specified

Table S5. The most commonly reported anatomical body locations amongst all reported injury complaints (*n*=1499), in sample of sporting and utility trial dogs (*n*=1582).

**Anatomical body location**	**Most common**	**Second most common**	**Third most common**	**Fourth most common**
Head	Tooth fracture. (n=41)	Traumatic injury, foreign body, dislocation, thermal injury. Conjunctiva, tear system, episclera, and cornea, sclera. (n=18)	Traumatic injury. Oral cavity, throat, esophagus. (n=4)	Wound. Skin, subcutaneous tissue, fur, feathers (n=2) Fracture. (n=2)
Neck	Herniated disc (n=5)	Pain. (n=3)	Fracture. (n=1) Muscle strain. (n=1) Spondylosis. (n=1) Traumatic injury. Non-organ specific. (n=1) Traumatic injury, foreign body, dislocation, thermal injury. Skeletal. (n=1)	N.A.
Thoracic, lumbar and lumbosacral spine	Spondylosis. (n=46)	Spinal dysfunction N.O.S. (n=40)	Herniated disc. (n=23)	Lumbosacral syndrome. (n=20)
Ribs or side	Sign of injury or disease N.O.S. Joint and ligament. (n=1)	N.A.	N.A.	N.A.
Pelvis	Fracture. (n=1)	Traumatic injury. Skeletal. (n=1)	Spinal issue from unspecified origin. (n=1)	N.A.
Tail	Acute Caudal Myopathy. (n=7)	Fracture. (n=3)	Ligament sprain, traumatic. (n=2)	Traumatic injury. Non-organ specific. (n=2) Altered mobility. Joint and ligament. (n=2)
CNS	Fibrocartilaginous embolism. Spinal cord, cauda equina. (n=2)	Brain concussion. (n=2)	N.A.	N.A.
PNS	Inflammation, infection. Peripheral nerves. (n=1)	Traumatic injury. Peripheral nerves. (n=1)	N.A.	N.A.
Shoulder	Muscle strain. (n=19)	Ligament sprain, traumatic. (n=7)	Osteochondrosis and/or osteochondrosis dissecans. (n=7)	Traumatic injury (including tendon rupture). Tendon, tendon sheath, bursa. (n=7) Metabolic, nutritional, degenerative/dystrophic changes. Tendon, tendon sheath, bursa. (n=7)
Brachium	Muscle strain. (n=6)	Myositits. (n=1)	N.A.	N.A.
Elbow	Fragmented medial coronoid process. (n=5)	Osteoarthritis. (n=5)	Traumatic injury. Non-organ specific. (n=4)	Inflammation. Joint and ligament. (n=2)
Antebrachium	Fracture. (n=1)	N.A.	N.A.	N.A.
Carpus	Ligament sprain, traumatic. (n=13)	Pain. (n=5)	Traumatic injury. Joint and ligament. (n=4)	N.A.
Forelimb N.O.S.	Lameness. (n=20)	Muscle strain. (n=6)	Pain. (n=5)	N.A.
Groin	Metabolic, nutritional, degenerative/dystrophic changes. Muscular. (M.m. iliopsoas) (n=9)	Muscle strain. (M.m. iliopsoas) (n=4)	N.A.	N.A.

Hip	Metabolic, nutritional, degenerative/dystrophic changes. Muscular. (n=6)	Osteoarthritis. (n=4)	Traumatic injury. Non-organ specific, and Traumatic injury. Joint and ligament. (n=3)	N.A.
Thigh	Muscle strain. MA.09.01.02. (n=20)	Wound. Skin, subcutaneous tissue, fur, feathers. HA.09.01.01. (n=2)	N.A.	N.A.
Stifle	Cranial Cruciate Ligament Rupture. (n=22)	Ligament sprain, traumatic. (n=8)	Patella luxation. (n=13)	Osteoarthritis. (n=6)
Tibial/fibular	Fracture. (n=3)	N.A.	N.A.	N.A.
Hock	Tendon luxation. (Superficial digital flexor tendon) (n=6)	Ligament sprain, traumatic. (n=4)	N.A.	N.A.
Hindlimb N.O.S.	Muscle strain. (n=25)	Lameness. (n=6)	Muscle Pain. (n=6)	Pain. (n=5)
Paw	Wound. Skin, subcutaneous tissue, fur, feathers. (n=32)	Puncture wound. Skin, subcutaneous tissue, fur, feathers. (n=9)	Ligament sprain, traumatic. (n=6)	Metabolic, nutritional, degenerative/dystrophic changes. Tendon, tendon sheath, bursa. (n=5)
Pad	Wound. Skin, subcutaneous tissue, fur, feathers. (n=59)	Puncture wound. Skin, subcutaneous tissue, fur, feathers. (n=4)	N.A.	N.A.
Digit	Fracture. (n=36)	Ligament sprain, traumatic. (n=26)	Metabolic, nutritional, degenerative/dystrophic changes. Tendon, tendon sheath, bursa. (n=18)	Osteoarthritis. (n=15)
Nail	Torn nail. (n=117)	N.A.	N.A.	N.A.
Limb N.O.S.	Wound. Skin, subcutaneous tissue, fur, feathers. (n=8)	Muscle strain. (n=7)	Metabolic, nutritional, degenerative/dystrophic changes. Tendon, tendon sheath, bursa (n=4)	Ligament sprain, traumatic. (n=3)
Bone N.O.S.	Fracture. (n=2)	Sesmoiditis. (n=1)	N.A.	N.A.
Joint N.O.S.	Osteoarthritis. (n=25)	Ligament sprain, traumatic. (n=18)	Osteochondrosis and/or osteochoondrosis dissecans. (n=7)	
Muscle N.O.S.	Muscle strain. (n=125)	Myositits. (n=11)	Contusion. Muscular. (n=4)	Muscle Pain. (n=4)
Pain N.O.S.	Lameness. (n=142)	Pain. (n=7)	N.A.	N.A.
Soft tissue N.O.S.	Wound. Skin, subcutaneous tissue, fur, feathers. (n=70)	Puncture wound. Skin, subcutaneous tissue, fur, feathers. (n=6)	N.A.	N.A.
Tendon N.O.S.	Metabolic, nutritional, degenerative/dystrophic changes. Tendon, tendon sheath, bursa. (n=4)	Tendon rupture. (n=2)	N.A.	N.A.
N.A. = Not applicable, N.O.S. = Not otherwise specified				

Table S6A. Injured body system, by disciplines, in a sample of Swedish sporting and utility trial dogs (*n*=1582) following sensitivity analysis excluding self-diagnosed injuries.

	**Full cohort**	**Obedience**	**Rally obedience**	**Agility**	**Utility**	**Protection**	**Tracking**	**Search**	**Messenger**
n of dogs participating	1582	790	596	430	847	169	667	226	33
n (%) of injured dogs	596 (37.7)	307 (38.9)	222 (37.2)	171 (39.8)	343 (40.5)	69 (40.8)	277 (41.5)	85 (37.6)	19 (57.6)
**Pyramidion diagnosis system**	***n* (%)**	***n* (%)**	***n* (%)**	***n* (%)**	***n* (%)**	***n* (%)**	***n* (%)**	***n* (%)**	***n* (%)**
Muscular	182 (11.5)	105 (13.3)	75 (12.6)	52 (12.1)	107 (12.6)	24 (14.2)	83 (12.4)	30 (13.3)	6 (18.2)
Joint and ligament	180 (11.4)	89 (11.3)	70 (11.7)	66 (15.3)	91 (10.7)	16 (9.5)	71 (10.6)	24 (10.6)	5 (15.2)
Skeletal	135 (8.5)	81 (10.3)	53 (8.9)	29 (6.7)	90 (10.6)	18 (10.7)	79 (11.8)	21 (9.3)	4 (12.1)
Dermatologic	128 (8.1)	67 (8.5)	52 (8.7)	35 (8.1)	70 (8.3)	16 (9.5)	58 (8.7)	19 (8.4)	5 (15.2)
Multiorgan	99 (6.3)	47 (5.9)	37 (6.2)	31 (7.2)	57 (6.7)	12 (7.1)	42 (6.3)	16 (7.1)	3 (9.1)
Ophthalmologic	12 (0.8)	4 (0.5)	4 (0.7)	2 (0.5)	6 (0.7)	1 (0.6)	4 (0.6)	2 (0.9)	1 (3.0)
Nervous	5 (0.3)	4 (0.5)	5 (0.8)	0 (0.0)	3 (0.4)	0 (0.0)	3 (0.4)	0 (0.0)	0 (0.0)
Digestion	31 (2.0)	19 (2.4)	11 (1.8)	6 (1.4)	21 (2.5)	6 (3.6)	17 (2.5)	6 (2.7)	2 (6.1)

Table S6B. Injured body system, by individual breeds and type, in a sample of Swedish sporting and utility trial dogs (*n*=1582) following a sensitivity analysis excluding self-diagnosed injuries.

	**Full cohort**	**Australian Kelpie**	**Australian Shepherd**	**Border Collie**	**Belgian Malinois**	**German Shepherd Dog**	**Labrador Retriever**	**Shetland Sheepdog**	**Other Purebreds**	**Mixed-breeds**
n of dogs participating	1582	76	86	133	111	205	67	67	791	46
n (%) of injured dogs	596 (37.7)	19 (25.0)	29 (33.7)	65 (48.9)	51 (45.9)	63 (30.7)	29 (43.3)	23 (34.3)	294 (37.2)	23 (50.0)
**Pyramidion diagnosis system**	***n* (%)**	***n* (%)**	***n* (%)**	***n* (%)**	***n* (%)**	***n* (%)**	***n* (%)**	***n* (%)**	***n* (%)**	***n* (%)**
Muscular	182(11.5)	5(6.6)	11(12.8)	23(17.3)	12(10.8)	19(9.3)	7(10.4)	9(13.4)	90(11.4)	6(13.0)
Joint and ligament	180(11.4)	8(10.5)	7(8.1)	29(21.8)	12(10.8)	18(8.8)	8(11.9)	10(14.99	81(10.2)	7(15.2)
Skeletal	135(8.5)	4(5.3)	8(9.3)	6(4.5)	10(9.0)	18(8.8)	6(9.0)	0(0.0)	80(10.1)	3(6.5)
Dermatologic	128(8.19	3(3.9)	8(9.3)	12(9.0)	14(12.6)	12(5.9)	11(16.4)	0(0.0)	63(8.0)	5(10.9)
Multiorgan	99(6.3)	4(5.3)	2(2.3)	14(10.5)	12(10.8)	9(4.4)	4(6.0)	4(6.0)	47(5.9)	3(6.5)
Ophthalmologic	12(0.8)	0(0.0)	0(0.0)	0(0.0)	1(0.9)	1(0.5)	3(4.5)	0(0.0)	7(0.9)	0(0.0)
Nervous	5(0.3)	1(1.3)	1(1.2)	0(0.0)	0(0.0)	0(0.0)	0(0.0)	0(0.0)	1(0.1)	2(4.3)
Digestion	31(2.0)	1(1.3)	4(4.7)	3(2.3)	6(5.4)	3(1.5)	2(3.0)	2(3.0)	10(1.3)	0(0.0)

‘

Table S7A. Injured anatomical location, by disciplines, in a sample of Swedish sporting and utility trial dogs (*n*=1582) following sensitivity analysis excluding self-diagnosed injuries.

	**Full cohort**	**Obedience**	**Rally obedience**	**Agility**	**Utility**	**Protection**	**Tracking**	**Search**	**Messenger**
n of dogs participating	1582	790	596	430	847	169	667	226	33
n (%) of injured dogs	596 (37.7)	307 (38.9)	222 (37.2)	171 (39.8)	343 (40.5)	69 (40.8)	277 (41.5)	85 (37.6)	19 (57.6)
**Anatomical body location**	***n* (%)**	***n* (%)**	***n* (%)**	***n* (%)**	***n* (%)**	***n* (%)**	***n* (%)**	***n* (%)**	***n* (%)**
Head	44 (2.8)	22 (2.8)	15 (2.5)	8 (1.9)	29 (3.4)	8 (4.7)	23 (3.4)	8 (3.5)	4 (12.1)
Neck	9 (0.6)	4 (0.5)	2 (0.3)	2 (0.5)	6 (0.7)	0 (0.0)	4 (0.6)	3 (1.3)	0 (0.0)
Thoracic, lumbar and lumbosacral spine	127 (8.0)	77 (9.7)	60 (10.1)	29 (6.7)	75 (8.9)	20 (11.8)	64 (9.6)	19 (8.4)	3 (9.1)
Pelvis	3 (0.2)	1 (0.1)	1 (0.2)	0 (0.0)	3 (0.4)	0 (0.0)	3 (0.4)	0 (0.0)	1 (3.0)
Tail	10 (0.6)	5 (0.6)	4 (0.7)	3 (0.7)	5 (0.6)	1 (0.6)	4 (0.6)	1 (0.4)	1 (3.0)
CNS	3 (0.2)	2 (0.3)	3 (0.5)	0 (0.0)	2 (0.2)	0 (0.0)	2 (0.3)	0 (0.0)	0 (0.0)
PNS	2 (0.1)	2 (0.3)	2 (0.3)	0 (0.0)	1 (0.1)	0 (0.0)	1 (0.1)	0 (0.0)	0 (0.0)
Shoulder	50 (3.2)	30 (3.8)	23 (3.9)	15 (3.5)	29 (3.4)	6 (3.6)	27 (4.0)	8 (3.5)	1 (3.0)
Brachium	4 (0.3)	3 (0.4)	2 (0.3)	1 (0.2)	4 (0.5)	1 (0.6)	4 (0.6)	2 (0.9)	1 (3.0)
Elbow	11 (0.7)	6 (0.8)	6 (1.0)	2 (0.5)	6 (0.7)	0 (0.0)	5 (0.7)	0 (0.0)	1 (3.0)
Antebrachium	1 (0.1)	1 (0.1)	0 (0.0)	0 (0.0)	1 (0.1)	0 (0.0)	1 (0.1)	0 (0.0)	0 (0.0)
Carpus	24 (1.5)	15 (1.9)	5 (0.8)	5 (1.2)	20 (2.4)	6 (3.6)	15 (2.2)	9 (4.0)	2 (6.1)
Forelimb	19 (1.2)	9 (1.1)	9 (1.5)	6 (1.4)	10 (1.2)	1 (0.6)	9 (1.3)	1 (0.4)	0 (0.0)
Groin	10 (0.6)	7 (0.9)	2 (0.3)	5 (1.2)	5 (0.6)	0 (0.0)	5 (0.7)	0 (0.0)	0 (0.0)
Hip	14 (0.9)	6 (0.8)	2 (0.3)	6 (1.4)	7 (0.8)	0 (0.0)	5 (0.7)	2 (0.9)	0 (0.0)
Thigh	12 (0.8)	5 (0.6)	2 (0.3)	2 (0.5)	11 (1.3)	5 (3.0)	8 (1.2)	5 (2.2)	1 (3.0)
Stifle	59 (3.7)	27 (3.4)	26 (4.4)	25 (5.8)	26 (3.1)	2 (1.2)	22 (3.3)	5 (2.2)	2 (6.1)
Tibial/fibular	2 (0.1)	2 (0.3)	0 (0.0)	0 (0.0)	2 (0.2)	0 (0.0)	2 (0.3)	1 (0.4)	0 (0.0)
Hock	12 (0.8)	5 (0.6)	7 (1.2)	7 (1.6)	4 (0.5)	1 (0.6)	4 (0.6)	1 (0.4)	0 (0.0)
Hindlimb	29 (1.8)	16 (2.0)	17 (2.9)	9 (2.1)	14 (1.7)	3 (1.8)	9 (1.3)	3 (1.3)	1 (3.0)
Paw	26 (1.6)	12 (1.5)	14 (2.3)	5 (1.2)	17 (2.0)	3 (1.8)	13 (1.9)	5 (2.2)	0 (0.0)

Pad	19 (1.2)	9 (1.1)	5 (0.8)	3 (0.7)	14 (1.7)	2 (1.2)	12 (1.8)	1 (0.4)	1 (3.0)
Digit	84 (5.3)	49 (6.2)	30 (5.0)	28 (6.5)	51 (6.0)	6 (3.6)	43 (6.4)	16 (7.1)	2 (6.1)
Nail	70 (4.4)	35 (4.4)	31 (5.2)	24 (5.6)	31 (3.7)	7 (4.1)	23 (3.4)	9 (4.0)	2 (6.1)
Limb N.O.S.	19 (1.2)	12 (1.5)	6 (1.0)	5 (1.2)	8 (0.9)	3 (1.8)	7 (1.0)	3 (1.3)	0 (0.0)
Bone N.O.S.	3 (0.2)	1 (0.1)	0 (0.0)	0 (0.0)	3 (0.4)	0 (0.0)	3 (0.4)	1 (0.4)	0 (0.0)
Joint N.O.S.	40 (2.5)	20 (2.5)	15 (2.5)	12 (2.8)	25 (3.0)	6 (3.6)	18 (2.7)	6 (2.7)	0 (0.0)
Muscle N.O.S.	59 (3.7)	29 (3.7)	18 (3.0)	18 (4.2)	37 (4.4)	8 (4.7)	26 (3.9)	9 (4.0)	1 (3.0)
Pain N.O.S.	25 (1.6)	13 (1.6)	8 (1.3)	8 (1.9)	10 (1.2)	2 (1.2)	6 (0.9)	4 (1.8)	1 (3.0)
Soft tissue N.O.S.	26 (1.6)	16 (2.0)	8 (1.3)	4 (0.9)	19 (2.2)	7 (4.1)	17 (2.5)	6 (2.7)	2 (6.1)
Tendon N.O.S.	4 (0.3)	3 (0.4)	2 (0.3)	0 (0.0)	4 (0.5)	1 (0.6)	4 (0.6)	1 (0.4)	0 (0.0)

N.O.S. = Not otherwise specified

Table S7B. Injured anatomical location, by individual breeds and type, in a sample of Swedish sporting and utility trial dogs (*n*=1582) following sensitivity analysis excluding self-diagnosed injuries.

	Full cohort	Australian Kelpie	Australian Shepherd	Border Collie	Belgian Malinois	German Shepherd Dog	Labrador Retriever	Shetland Sheepdog	Other Purebreds	Mixed Breeds
n of dogs participating	1582	76	86	133	111	205	67	67	791	46
n (%) of injured dogs	596 (37.7)	19 (25.0)	29 (33.7)	65 (48.9)	51 (45.9)	63 (30.7)	29 (43.3)	23 (34.3)	294 (37.2)	23 (50.0)
**Anatomical body location**	***n* (%)**	***n* (%)**	***n* (%)**	***n* (%)**	***n* (%)**	***n* (%)**	***n* (%)**	***n* (%)**	***n* (%)**	***n* (%)**
Head	44 (2.8)	1 (1.3)	4 (4.7)	3 (2.3)	7 (6.3)	6 (2.9)	5 (7.5)	2 (3.0)	16 (2.0)	0 (0.0)
Neck	9 (0.6)	0 (0.0)	0 (0.0)	0 (0.0)	1 (0.9)	0 (0.0)	0 (0.0)	0 (0.0)	8 (1.0)	0 (0.0)
Thoracic, lumbar, lumbosacral spine	127 (8.0)	5 (6.6)	7 (8.1)	9 (6.8)	9 (8.1)	21 (10.2)	3 (4.5)	2 (3.0)	65 (8.2)	6 (13.0)
Pelvis	3 (0.2)	0 (0.0)	0 (0.0)	0 (0.0)	1 (0.9)	0 (0.0)	0 (0.0)	0 (0.0)	2 (0.3)	0 (0.0)
Tail	10 (0.6)	1 (1.3)	0 (0.0)	2 (1.5)	0 (0.0)	1 (0.5)	0 (0.0)	0 (0.0)	6 (0.8)	0 (0.0)
CNS	3 (0.2)	1 (1.3)	1 (1.2)	0 (0.0)	0 (0.0)	0 (0.0)	0 (0.0)	0 (0.0)	0 (0.0)	1 (2.2)
PNS	2 (0.1)	0 (0.0)	0 (0.0)	0 (0.0)	0 (0.0)	0 (0.0)	0 (0.0)	0 (0.0)	1 (0.1)	1 (2.2)
Shoulder	50 (3.2)	4 (5.3)	2 (2.3)	9 (6.8)	3 (2.7)	5 (2.4)	2 (3.0)	1 (1.5)	23 (2.9)	1 (2.2)
Brachium	4 (0.3)	0 (0.0)	0 (0.0)	0 (0.0)	1 (0.9)	0 (0.0)	0 (0.0)	0 (0.0)	3 (0.4)	0 (0.0)
Elbow	11 (0.7)	0 (0.0)	0 (0.0)	1 (0.8)	0 (0.0)	1 (0.5)	2 (3.0)	0 (0.0)	6 (0.8)	1 (2.2)
Antebrachium	1 (0.1)	0 (0.0)	0 (0.0)	0 (0.0)	0 (0.0)	0 (0.0)	0 (0.0)	0 (0.0)	1(0.1)	0 (0.0)
Carpus	24 (1.5)	3 (3.9)	0 (0.0)	2 (1.5)	5 (4.5)	4 (2.0)	0 (0.0)	1 (1.5)	8 (1.0)	1 (2.2)
Forelimb	19 (1.2)	1 (1.3)	1 (1.2)	1 (0.8)	1 (0.9)	1 (0.5)	1 (1.5)	1 (1.5)	10 (1.3)	2 (4.3)
Groin	10 (0.6)	0 (0.0)	1 (1.2)	1 (0.8)	0 (0.0)	0 (0.0)	0 (0.0)	1 (1.5)	7 (0.9)	0 (0.0)
Hip	14 (0.9)	0 (0.0)	1 (1.2)	2 (1.5)	1 (0.9)	2 (1.0)	0 (0.0)	2 (3.0)	6 (0.8)	0 (0.0)
Thigh	12 (0.8)	1 (1.3)	1 (1.2)	0 (0.0)	0 (0.0)	6 (2.9)	0 (0.0)	0 (0.0)	3 (0.4)	1 (2.2)
Stifle	59 (3.7)	2 (2.6)	5 (5.8)	7 (5.3)	3 (2.7)	2 (1.0)	4 (6.0)	1 (1.5)	31 (3.9)	4 (8.7)
Tibial/fibular	2 (0.1)	0 (0.0)	0 (0.0)	0 (0.0)	0 (0.0)	0 (0.0)	0 (0.0)	0 (0.0)	2 (0.3)	0 (0.0)
Hock	12 (0.8)	0 (0.0)	0 (0.0)	1 (0.8)	1 (0.9)	1 (0.5)	0 (0.0)	6 (9.0)	3 (0.4)	0 (0.0)
Hindlimb	29 (1.8)	0 (0.0)	2 (2.3)	7 (5.3)	3 (2.7)	2 (1.0)	1 (1.5)	0 (0.0)	13 (1.6)	1 (2.2)
Paw	26 (1.6)	0 (0.0)	2 (2.3)	0 (0.0)	1 (0.9)	5 (2.4)	2 (3.0)	1 (1.5)	15 (1.9)	0 (0.0)

Pad	19 (1.2)	0 (0.0)	1 (1.2)	2 (1.5)	0 (0.0)	3 (1.5)	0 (0.0)	0 (0.0)	12 (1.5)	1 (2.2)
Digit	84 (5.3)	4 (5.3)	5 (5.8)	13 (9.8)	8 (7.2)	3 (1.5)	7 (10.4)	1 (1.5)	43 (5.4)	0 (0.0)
Nail	70 (4.4)	2 (2.6)	5 (5.8)	6 (4.5)	6 (5.4)	3 (1.5)	7 (10.4)	0 (0.0)	38 (4.8)	3 (6.5)
Limb N.O.S.	19 (1.2)	0 (0.0)	0 (0.0)	5(3.8)	1 (0.9)	1 (0.5)	2 (3.0)	2 (3.0)	6 (0.8)	2 (4.3)
Bone N.O.S.	3 (0.2)	0 (0.0)	0 (0.0)	0 (0.0)	0 (0.0)	0 (0.0)	0 (0.0)	0 (0.0)	3 (0.4)	0 (0.0)
Joint N.O.S.	40 (2.5)	0 (0.0)	3 (3.5)	5 (3.8)	2 (1.8)	7 (3.4)	2 (3.0)	4 (6.0)	17 (2.1)	0 (0.0)
Muscle N.O.S.	59 (3.7)	1 (1.3)	3 (3.5)	9 (6.8)	5 (4.5)	7 (3.4)	4 (6.0)	1 (1.5)	27 (3.4)	2 (4.3)
Pain N.O.S.	25 (1.6)	2 (2.6)	0 (0.0)	2 (1.5)	3 (2.7)	1 (0.5)	2 (3.0)	0 (0.0)	14 (1.8)	1 (2.2)
Soft tissue N.O.S.	26 (1.6)	0 (0.0)	1 (1.2)	2 (1.5)	7 (6.3)	4 (2.0)	2 (3.0)	0 (0.0)	10 (1.3)	0 (0.0)
Tendon N.O.S.	4 (0.3)	0 (0.0)	0 (0.0)	1 (0.8)	0 (0.0)	0 (0.0)	0 (0.0)	0 (0.0)	3 (0.4)	0 (0.0)
N.O.S. = Not otherwise specified										
